# A Straightforward Access to Sustainable and Reusable Melanin-Supported Palladium Catalysts: Characterization and Application in Sonogashira Cross-Coupling Reactions

**DOI:** 10.1021/acsami.5c23680

**Published:** 2026-03-31

**Authors:** Simone Potenti, Pasquale Lioniello, Elisabetta Mazzotta, Nicoletta Ditaranto, Evelina Liarou, Noah Al-Shamery, Pooi See Lee, Teresa Sibillano, Cinzia Giannini, Cosimino Malitesta, Paola Manini

**Affiliations:** † Department of Chemical Sciences, University of Naples Federico II, via Cintia 4, I-80126 Naples, Italy; ‡ Bioelectronics Task Force, University of Naples Federico II, via Cintia 4, I-80126 Naples, Italy; § Dipartimento di Scienze e Tecnologie Biologiche e Ambientali (Di.S.Te.B.A.), 18976 Università del Salento, via Monteroni 165, I-73100 Lecce, Italy; ∥ Dipartimento di Chimica and CSGI − Bari Unit, 9295 Università degli Studi di Bari Aldo Moro, Via Orabona 4, I-70126 Bari, Italy; ⊥ Department of Chemistry, 2707 University of Warwick, Library Road, CV4 7AL Coventry, United Kingdom; # School of Materials Science and Engineering, 54761 Nanyang Technological University, 50 Nanyang Ave, Singapore 639798, Singapore; ∇ Institute of Crystallography (CNR-IC), National Council of Research, Via G. Amendola 122/O, I-70126 Bari, Italy

**Keywords:** melanin, palladium supported catalysts, sustainable catalysis, hybrid materials, cross-coupling reaction

## Abstract

In the last decades, the impact of the emergent concept of sustainable development has driven the impressive evolution of catalytic methodologies, also stimulated by the breakthrough of new materials and nanotechnologies. Based on this evidence, we report herein the design of sustainable catalysts by addressing the unexploited potential of melanin, a class of pigments widely diffused in nature. By pursuing a straightforward and sustainable approach, we prepared a series of Pd@melanin hybrids inspired from mammalian eumelanin and fungal allomelanin. XPS analyses revealed that DHImel (DHI: 5,6-dihydroxyindole) can support palladium by promoting the reduction of Pd­(II) to Pd(0) thanks to the catechol groups that can be converted into quinones. HR-TEM and XRD analyses revealed that palladium atoms are uniformly distributed, forming nanoaggregates in the face-centered cubic crystal structure with an average dimension of 8 nm. The catalytic activity of the hybrids was tested in the Sonogashira cross-coupling reaction. Overall, the collected data suggested that Pd@DHImel, with the highest product yields and lowest Pd loading, is the most efficient catalyst, even when working with deactivated aromatic iodides. Finally, stability tests indicated that the hybrid did not suffer leaching phenomena, leading to high yields for up to eight cycles.

## Graphical abstract

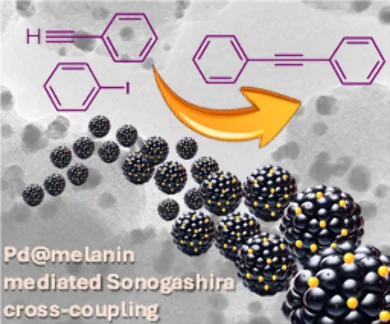

## 1 Introduction

Since the birth of catalysis as an essential research field, increasing efforts—both experimental and theoretical—have been devoted to the development of new catalysts. Over time, many different urgent needs were considered, such as the development of new processes, the availability of different starting materials, and, more recently, the increasing pressure to meet sustainability requirements.^1,2^ In this context, the intimate connection between catalysis and bioinspired processes is now well rooted in the scientific community.^3^ A valuable example comes from the application of the concepts of the directed evolution theory, not only affecting the field of biocatalysis but also boosting the design of more powerful and selective artificial enzymes.^4,5^

In the context of sustainability, interesting results have been obtained by using catalysts derived from agrifood waste materials as a pivotal strategy in the framework of a circular economy. Indeed, many important waste materials such as lignin, cellulose, and chitosan are currently studied as precious starting materials in the field of catalysis.^6–8^

Although top-down approaches leading to catalytic materials from complex wastes are very appealing, the bottom-up approaches from commercially available simple starting materials ensure low batch-to-batch variability; in the latter case, sustainability principles are highly desired even for their synthesis, limiting the use of hazardous organic solvents in favor of aqueous media. In this context, melanin represents an intriguing class of polymers that meets many of the aforementioned requirements. Melanin consists of a wide group of pigments differing for composition, occurrence, and functions.^9^ They are found in mammals (eumelanin, pheomelanin, and neuromelanin), in plants and fungi (allomelanin),^10^ and in bacteria (pyomelanin). From a biosynthetic point of view, all melanin species are formed via the oxidative polymerization of phenolic derivatives. Melanin can be isolated from natural sources,^11^ including hairs, iris, the ink sack of cuttlefish, insect cuticles,^12^ and certain types of fungi and bacteria.^13^ Moreover, they can be synthesized starting from commercially available precursors such as 3,4-dihydroxyphenylalanine (DOPA), dopamine (DA), 5,6-dihydroxyindole (DHI) and its 2-carboxylic acid derivative (DHICA), and 1,8-dihydroxynaphtalene (DHN)^11,14^ by using mild protocols under environmental benign conditions. The uniqueness of melanin with respect to other biopolymers (i.e., cellulose, chitosan) relies on the combination of a series of chemical-physical properties that in the last decades has attracted particular interest for the design of sustainable functional biomaterials. Melanin are insoluble and biocompatible polymers characterized by an intrinsic microporosity and an unusual chemical and thermal robustness.^15^ They exhibit a redox behavior driven by the catecholic/phenolic groups accounting for the marked antioxidant power, an intrinsic paramagnetic character, a broadband absorption over the entire UV–visible spectrum responsible for the dark coloration, a hybrid ionic/electronic conductivity, and good metal chelating properties (Figure 1).^16,17^

**Figure 1:**
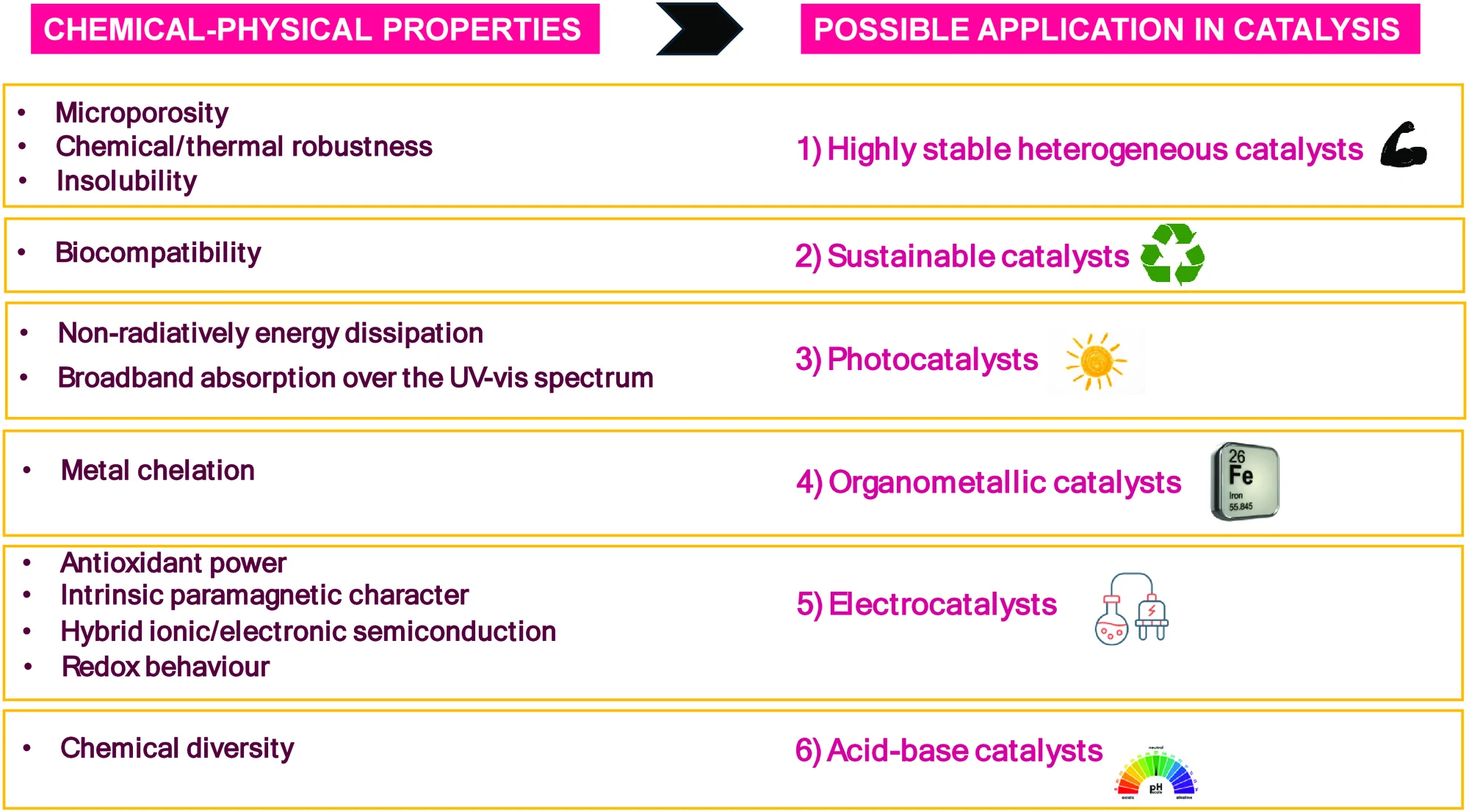
Chemical-physical properties of melanin and their relevance for the design of catalysts.

Taken together, all of these properties seem to open up the design of a new class of multifunctional catalysts whose activity can be tuned or combined according to the reaction conditions. In this connection, preliminary studies have shown how melanin can act as valuable photocatalysts in the reductive dehalogenation of α-halogenated carbonyl compounds.^18^ The results denoted a different chemical behavior among the melanin catalysts, probably ascribable to the different structural features of the polymers. Moreover, DHI-melanin thin films, when irradiated in aqueous solutions with simulated solar light, proved to be able to reduce oxygen to hydrogen peroxide with the concomitant oxidation of suited sacrificial agents such as oxalate, formate or phenol, thus paving the way to the possible use of these pigments in photo­(electro)­catalysis.^19^

By pursuing a different approach, some melanin polymers, such as polydopamine or DOPA-melanin, have been used successfully to prepare Pd-based catalysts that have been used to promote Suzuki cross-coupling reactions.^19,20^ Their metal-binding ability can prevent the aggregation of metal nanoparticles by providing stabilization to the metal centers. Moreover, thanks to the presence of numerous catechol sites, melanin polymers can interact with Pd­(II) ions, promoting their reduction to Pd(0) without the addition of reducing agents, thus allowing the use of commercially available water-soluble salts as palladium sources.

Prompted by this evidence, we decided to explore the potential role of different types of melanin as a sustainable support for catalytically active palladium species. The interest in the design of Pd supported catalysts is driven by the need for low-cost materials with high atom efficiency and stability coupled with good catalytic performance. On this basis, numerous studies have been reported in the literature dealing with the selection of different types of support including inorganic (i.e., silica, zeolites),^21,22^ metal–organic (i.e., MOFs), and organic (i.e., polymers, carbon nanomaterials)^23,24^ materials. Recently, prompted by the urgent need for sustainable processes, the attention has been moved to the use of natural supports, including biopolymers (i.e., cellulose, silk fibroin) or waste materials, which are abundant, low-cost, and nontoxic.^25,26^

Based on these premises, in this work we report on the synthesis and characterization of new Pd@melanin hybrid materials starting from the melanin polymers obtained via the oxidative polymerization of DHI and DHICA, as synthetic mimics of eumelanin, and of DHN, as a synthetic mimic of fungal allomelanin. Later, we tested the catalytic performances of the Pd@melanin hybrids in the Sonogashira cross-coupling reaction, a highly effective method for building C–C bonds by using activated aryl derivatives and terminal alkynes as reaction partners.

The use of palladium catalysts supported on natural polymers is widely documented in the literature,^25,27,28^ denoting the high attention paid to this sustainable solution for running the Sonogashira cross-coupling reaction. On this basis, in this work, the efficiency of the Pd@melanin catalysts will be discussed and compared with those from other natural Pd-based catalysts.

## 2 Experimental Section

### 2.1 Materials

Phenylacetylene, 1,8-dihydroxynaphthalene, trimethylsilylacetylene, *p*-iodoanisole, *p*-iodotoluene, *p*-iodonitrobenzene, sodium ascorbate, horseradish peroxidase (Type II), and hydrogen peroxide (30% w/w water solution) were purchased from Sigma-Aldrich and used as received. 5,6-Dihydroxyindole and 5,6-dihydroxyindole-2-carboxylic acid were prepared following procedures available in the literature.^11,29^ Lacey carbon supported Cu TEM grids were purchased from Agar Scientific.

### 2.2 Characterization

#### 2.2.1 X-ray Powder Diffraction (XRPD) Analysis

XRPD patterns were collected using a Rigaku RINT2500 rotating anode diffractometer (50 kV, 200 mA) equipped with a Rigaku D/teX Ultra detector. An asymmetric Johansson Ge(111) crystal was used to select monochromatic Cu K_α1_ radiation (λ = 1.54056 Å). Measurements were executed in transmission mode by introducing the sample into a Lindemann glass capillary tube with a diameter of 0.5 mm. The XRPD patterns were recorded in the 2θ range of 10–120° for all samples, using 2θ increments of 0.02° and a fixed counting time of 2 s per step.

#### 2.2.2 X-ray Photoelectron Spectroscopy (XPS)

X-ray photoelectron spectroscopy (XPS) measurements were performed with a Versa Probe II Scanning XPS Microprobe spectrometer (Physical Electronics GmbH, Ismaning, Germany) using a monochromatized Al K_α_ source with an X-ray spot size of 200 mm and a power of 47.6 W. Wide scans and detailed spectra were acquired in fixed analyzer transmission mode with a pass energy of 117.40 and 29.35 eV and a step size of 1.0 and 0.1 eV, respectively. An electron gun was used for charge compensation (1.0 V, 20.0 mA). Spectral analysis was performed using CasaXPS software (version 2.3.16, Casa Software, Ltd., Teignmouth, UK). For the analysis of high-resolution spectra all peaks were fitted using Shirley background and GL(30) line shape (a combination of Gaussian 70% and Lorentzian 30%). For quantitative analysis, the relative sensitivity factors present in the library of CasaXPS were used for the areas of the signals. Surface charging was corrected considering adventitious C 1s (binding energy (BE) = 285.0 eV).

#### 2.2.3 Transmission Electron Microscopy (TEM) and Energy-Dispersive X-ray (EDX) Spectroscopy

TEM imaging was carried out using a JEOL 2100 LaB6 electron microscope at an accelerating voltage of 200 kV. The samples were prepared at concentrations of 1.5 mg·mL^–1^ in ethanol, with aliquots of 5 μL being drop casted on lacey carbon supported copper TEM grids. The samples were left to dry at ambient conditions for 24 h before imaging. EDX was performed in the same instrument using an Oxford Instruments silicon drift detector (SDD).

#### 2.2.4 Annular Dark Field–Scanning Transmission Electron Microscopy (ADF-STEM)

ADF-STEM images were acquired using a probe and image aberration corrected JEOL ARM200F TEM operating at 200 kV, equipped with a Schottky field emission gun (FEG) and a Gatan Orius SC1000 CCD camera, with a camera length of 8 cm, probe current of ∼23 pA, and semiconvergence angle of ∼25 mrad. The samples were prepared at concentrations of 1.5 mg·mL^–1^ in ethanol, with aliquots of 5 μL being drop casted on lacey carbon supported copper TEM grids. Prior to imaging, the prepared grids were placed in a vacuum at 40 °C for 24 h.

### 2.3 Synthesis of Melanin Polymers: General Procedure

A 10 mM solution of the proper substrate (DHI, DHN or DHICA, 200 mg) in aqueous phosphate buffer (0.1 M, pH = 7) was treated under vigorous stirring with horseradish peroxidase (HRP, 15 U/mL) and H_2_O_2_ (1.2 equiv). After 24 h, the mixture was treated with HCl 2 M until the pH was 3. The resulting precipitate was collected by centrifugation (7000 rpm for 15 min at 4 °C), washed three times with water, and freeze-dried.

### 2.4 Synthesis of Pd@Melanin Hybrids

A suspension of the melanin polymer (20 mg) in water (7 mL) was treated with a solution of Pd­(OAc)_2_ (13.5 mg (L-series), 135 mg (H-series)) in acetone (7 mL). The reaction mixture was kept at 90 °C under vigorous stirring for 3 h and then was centrifuged (7000 rpm for 15 min at 4 °C). The solid was washed twice with water, collected, and then freeze-dried to get the final powder.

### 2.5 Sonogashira Cross-Coupling Reaction: General Procedure

A solution of the aryl halide (0.19 mmol) in toluene (1.6 mL) was purged with an argon flux and treated with PPh_3_ (0.019 mmol), CuI (0.019 mmol), and the proper amount of the Pd@melanin catalyst. Then trimethylsilylacetylene or phenylacetylene (0.19 mmol) was added and the reaction was started with the addition of triethylamine (1.6 mL). The reaction mixture was kept at 60 °C under stirring for 1 h, then was centrifuged (7000 rpm for 15 min at 4 °C) to remove the solid catalyst. This latter was washed extensively with ethyl acetate, methanol, and acetone to allow for a quantitative recovery of the products, and the supernatant was extracted twice with a water solution of NH_4_Cl (10% w/w). The organic layers were collected, dried over anhydrous sodium sulfate and taken to dryness at reduced pressure. The reaction yields were evaluated by registering ^1^H NMR spectra of the residue dissolved in CDCl_3_. In the case of reuse of the catalyst, after 1 h reaction time the mixture was centrifuged (7000 rpm for 15 min at 4 °C) to separate the solid from the supernatant. This was then worked up as reported above, whereas the solid was washed with acetone, ethyl acetate, and methanol, freeze-dried, and used for a new reaction.

## 3 Results and Discussion

### 3.1 Synthesis and Characterization of Pd@Melanin Hybrids

To explore the potential of the melanin polymers in the formation of the palladium hybrids, three different types of synthetic pigments were prepared according to procedures reported in the literature.^11,14^ In particular, DHImel and DHICAmel, inspired from the natural mammalian eumelanin, and DHNmel, inspired from the natural fungal allomelanin, were obtained through the oxidative polymerization of DHI, DHICA, and DHN, respectively. This choice was made considering that DHImel, DHICAmel, and DHNmel exhibit different structural features that may drive different interactions with palladium atoms (Figure 2). In particular, DHNmel is a nitrogen-free pigment exhibiting phenolic moieties, whereas DHImel and DHICAmel are nitrogen-containing pigments characterized by the presence of catechol functionalities and, in the case of DHICAmel, carboxyl groups. As widely reported in the literature,^30,31^ the melanin structure has not been delineated for the difficulties associated with the insoluble and amorphous character; however, it is known that different levels of disorder act on the mode of polymerization, the supramolecular assembly, and the redox asset, shaping the properties of melanin (Figure 2).

**Figure 2:**
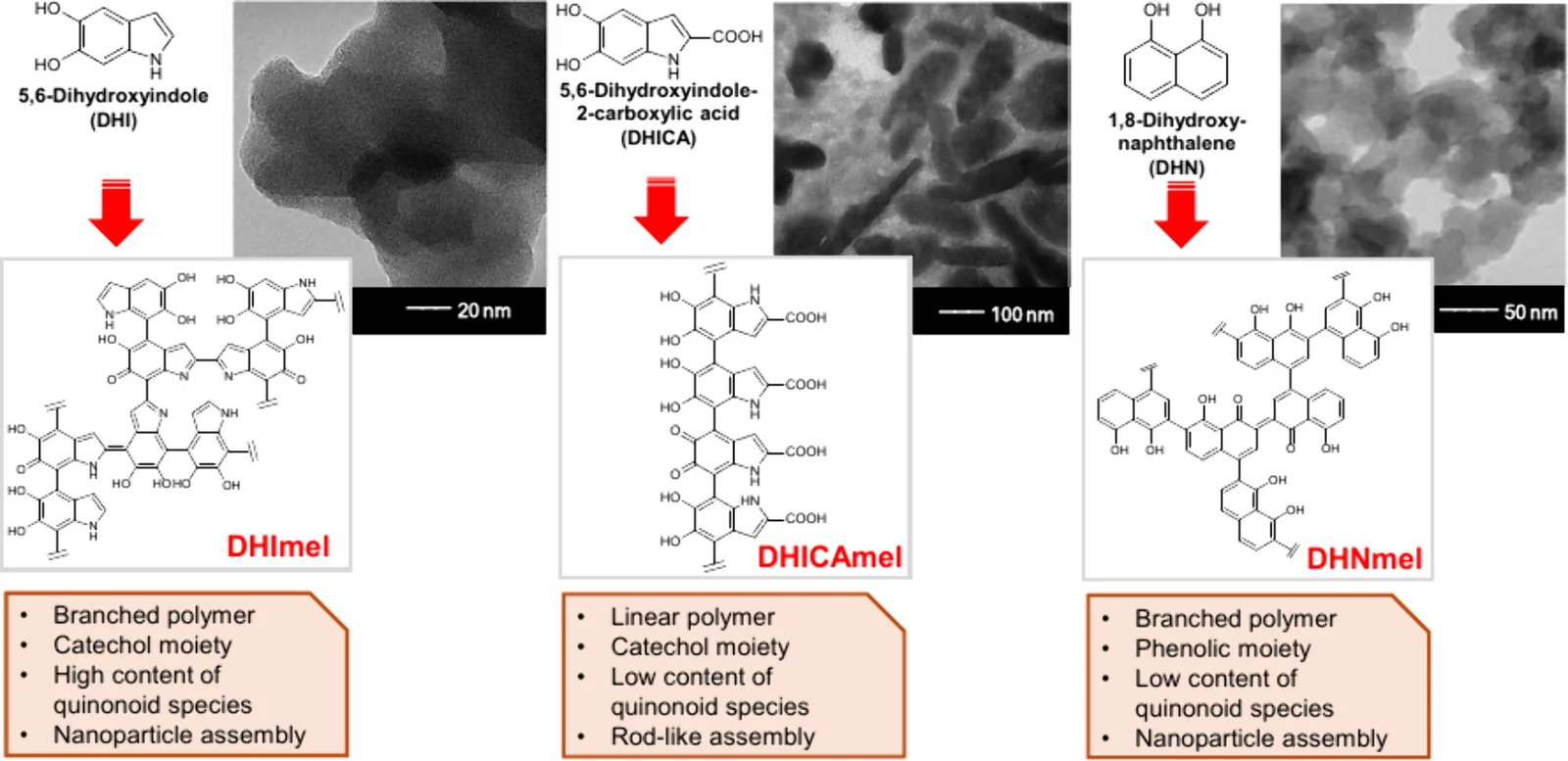
DHImel, DHICAmel, and DHNmel: proposed structures, main features, TEM images, and monomer precursors.

The protocols for the synthesis of melanin polymers rely on straightforward and sustainable processes. Indeed, they usually require a single step, with neither workup nor a purification step needed, and the melanin polymers are easily recovered as solid materials by centrifugation. Moreover, the typical reaction is an oxidative polymerization promoted by the enzymatic system horseradish peroxidase/hydrogen peroxide, to be performed in aqueous media (Figure 3). Similar considerations also apply to the preparation of Pd@melanin hybrids. While other carbon-based nanostructures usually require harsh conditions to afford the Pd hybrids (i.e., hydrothermal treatment, pyrolysis, argon glow discharge plasma), Pd@melanin hybrids were prepared by using mild conditions: the melanin polymers were suspended in water and treated with a solution of palladium acetate in acetone for 3 h at 90 °C. Thanks to the high insolubility of melanin, the final hybrids could be recovered by centrifugation with neither workup nor purification steps needed.

**Figure 3:**
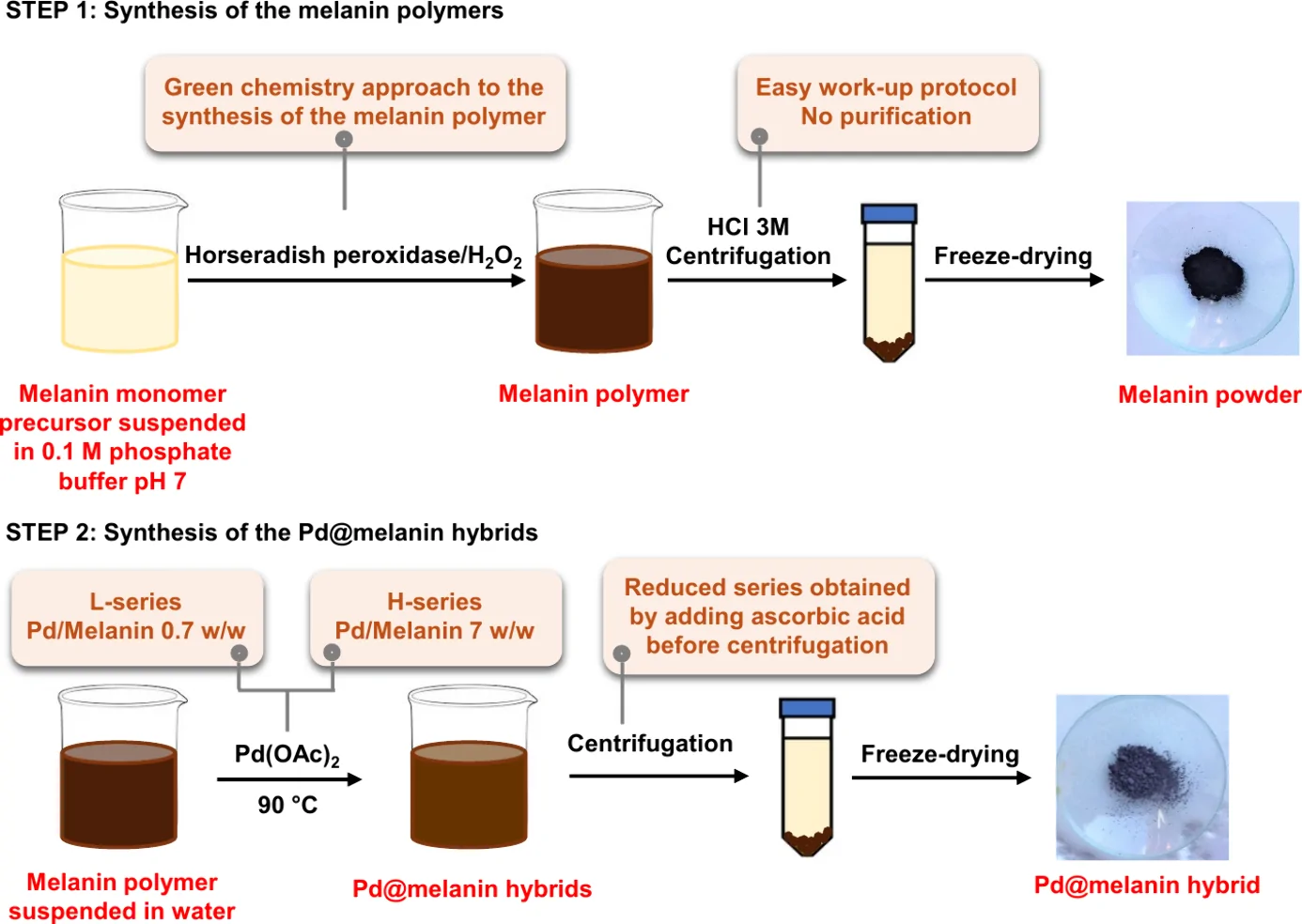
Synthetic protocols for the preparation of the melanin polymers and the corresponding Pd@melanin hybrids.

By pursuing this approach, a set of Pd@melanin catalysts (Table 1) were prepared while varying the following conditions: (1) the nature of the melanin polymer (Pd@DHImel, Pd@DHICAmel, Pd@DHNmel); (2) the palladium/melanin ratio ranging from 7 w/w (H-series) to 0.7 w/w (L-series); and (3) the additional reducing treatment to enhance the conversion of Pd­(II) to Pd(0) upon addition of ascorbic acid to the reaction mixture before the isolation of the hybrid (red series).

**Table 1:** Pd@Melanin Hybrids Prepared and Tested in This Work

Pd@melanin hybrid	melanin type	palladium/melanin ratio (w/w)	reduction treatment
Pd@DHImel_L	DHImel	0.7	-
Pd@DHImel_Lred	DHImel	0.7	ascorbic acid
Pd@DHICAmel_L	DHICAmel	0.7	-
Pd@DHICAmel_Lred	DHICAmel	0.7	ascorbic acid
Pd@DHNmel_L	DHNmel	0.7	-
Pd@DHNmel_Lred	DHNmel	0.7	ascorbic acid
Pd@DHImel_H	DHImel	7	-
Pd@DHImel_Hred	DHImel	7	ascorbic acid
Pd@DHICAmel_H	DHICAmel	7	-
Pd@DHICAmel_Hred	DHICAmel	7	ascorbic acid
Pd@DHNmel_H	DHNmel	7	-
Pd@DHNmel_Hred	DHNmel	7	ascorbic acid

Once obtained, all of the Pd@melanin hybrids were subjected to extensive characterization. The first evidence of palladium incorporation into the melanin polymers was provided by an X-ray diffraction (XRD) analysis. As shown in Figure 4, all the hybrid samples display a diffraction pattern resulting from the superposition of the broad signal, characteristic of amorphous melanin polymers (centered at approximately 2θ ≈ 24°),^32,33^ and sharper reflections corresponding to crystalline palladium in the face-centered cubic (fcc) crystal structure (space group *Fm* 3 *m*). Additional peaks indicative of the presence of crystalline phases were observed in the case of the Pd@DHICAmel sample. Qualitative analysis identified them as sodium sulfite (Na_2_SO_3_), sodium thiosulfate (Na_2_S_2_O_3_), and elemental sulfur (S), likely formed as byproducts during the synthetic process (Figure S3). Importantly, no reflections attributable to unreacted palladium acetate (Pd_3_(OAc)_6_) were detected in any of the samples, suggesting complete conversion of the precursor during synthesis.

**Figure 4:**
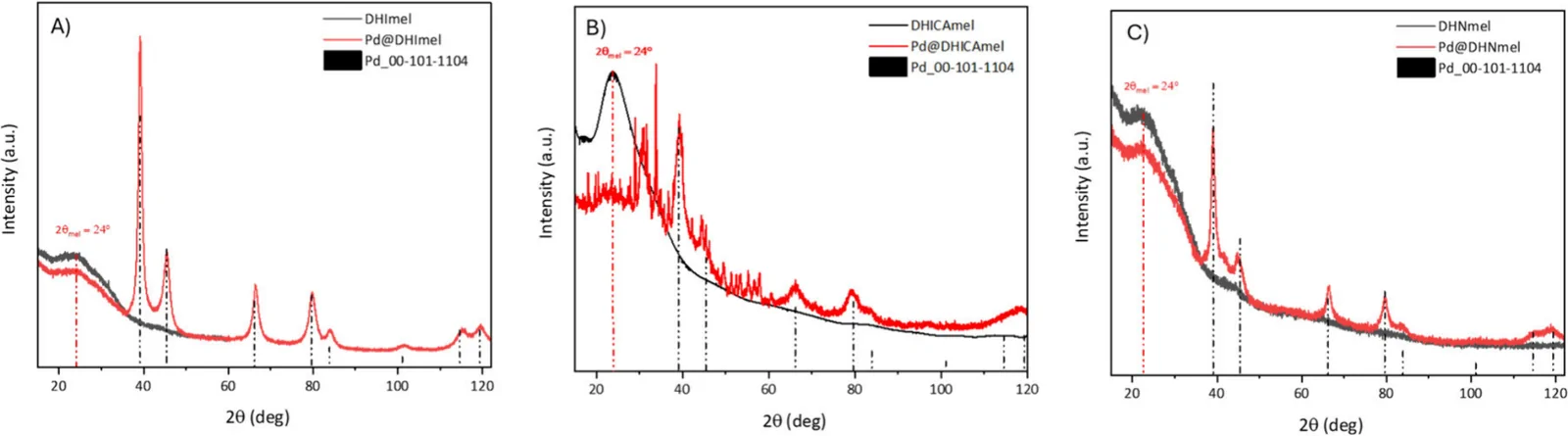
X-ray diffraction patterns of Pd@melanin hybrids, each compared with the bare melanin polymer. (A) Pd@DHImel and DHImel; (B) Pd@DHICAmel and DHICAmel; (C) Pd@DHNmel and DHNmel.

To further investigate the crystalline Pd domains, both lattice parameter refinement and apparent crystallite size estimation were carried out. The XRD patterns were analyzed using a whole-profile Rietveld-based fitting procedure implemented in the FULLPROF suite, assuming the fcc crystal structure for metallic palladium.

For the Pd@DHImel sample, Rietveld refinement yielded a cubic cell parameter of *a* = *b* = *c* = 3.98 Å, with an apparent crystallite size of 64.7 ± 13.3 Å (Figure S1). For the Pd@DHNmel sample, the refined lattice parameter was *a* = *b* = *c* = 3.99 Å and the apparent crystallite size was estimated to be 59.9 ± 15.6 Å (Figure S2). These values are consistent with the structural features of nanocrystalline palladium in the fcc crystal structure. The estimated domain sizes (6–7 nm) correspond to roughly 15–16 unit cells, supporting the formation of well-dispersed Pd nanoparticles embedded within the hybrid matrix.

The presence of Pd was also verified via HR-TEM analysis (Figure 5) supported by energy dispersive X-ray spectroscopy (EDX), by comparing a Pd-rich region with a corresponding organic-content-rich area of the sample without the presence of Pd species (Figure S4).

**Figure 5:**
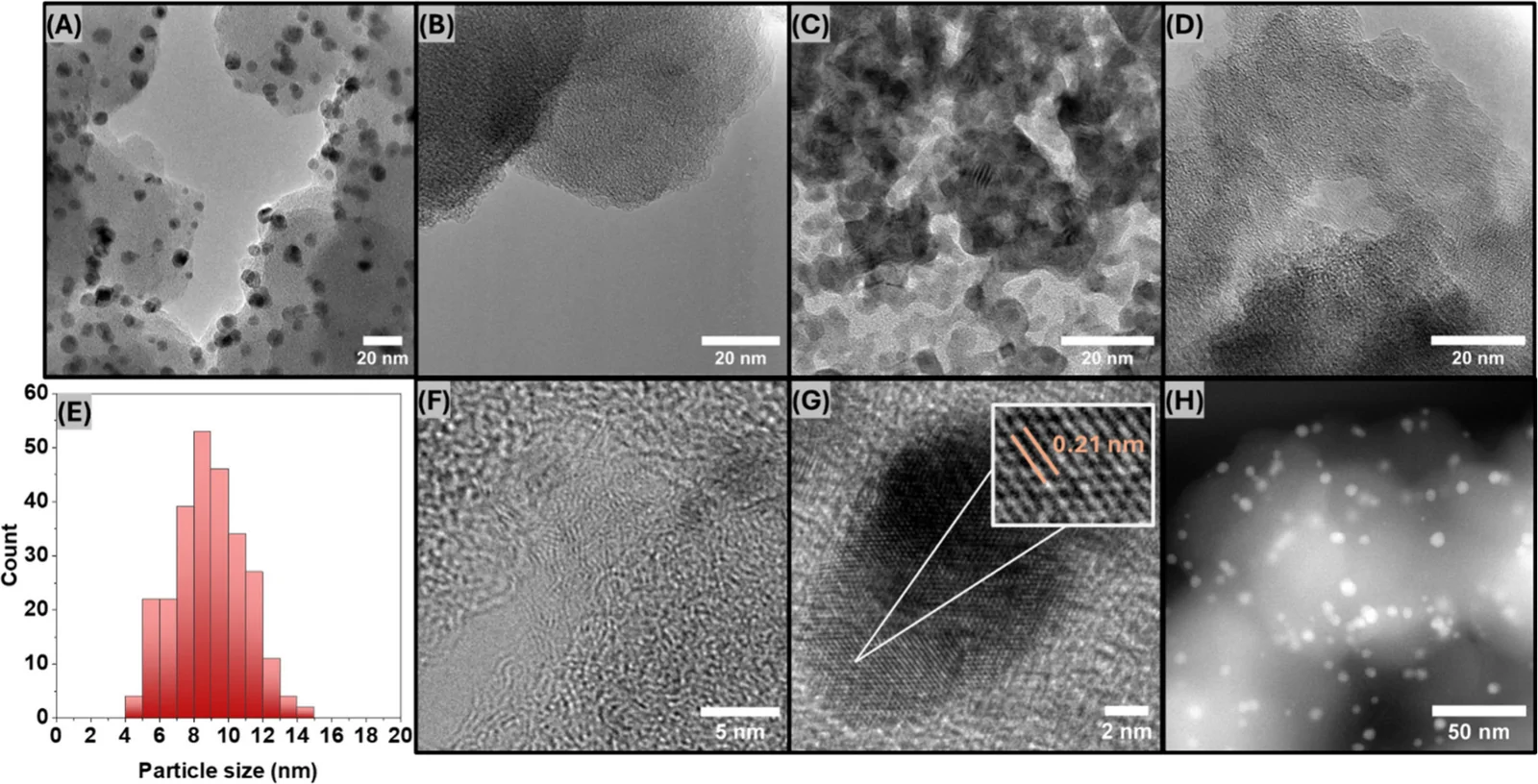
HR-TEM images from Pd@melanin hybrids compared with the respective bare melanin polymer. Pd@DHImel L-series (A); DHImel (B); Pd@DHNmel H-series (C); and DHNmel (D). Pd@DHImel L-series: palladium average particle size (E) and spacing features of the organic (F) and Pd (G) regions. Annular dark field–scanning transmission electron microscopy (ADF-STEM) image of the Pd@DHImel L-series (H).

As anticipated from the XRD data, HR-TEM images revealed that palladium incorporation did not affect the morphology of the melanin polymers. Spacing features of the organic part that are indicative of melanin’s structural arrangements (Figure 5F) can be observed that exhibit distinctive differences from the well-distributed Pd species with distinctive lattice spacing features (0.21 nm) (Figure 5G).^23^

A relatively homogeneous distribution of the metal over the melanin polymer was evident from the inspection of the HR-TEM images of hybrids from the L-series (Figure 5A), with well-dispersed nanoparticles of metallic palladium detectable (nanoparticle average diameter = 8.5 nm; Figure 5E). In contrast, a less homogeneous distribution with more evident Pd aggregates can be detected in the hybrids of the H-series due to the agglomeration caused by a high metal loading (Figure 5C).

The uniform Pd dispersion in the hybrids of the L-series was also confirmed by annular dark field–scanning transmission electron microscopy (ADF-STEM), with the well-dispersed Pd clusters appearing bright owing to the high Z-contrast of Pd (Figure 5H).

XPS analyses were carried out to check for the loading of the metal onto melanin and to address the nature of the interactions between the metal and the polymer. Significant differences emerged among the three melanin types, with DHImel showing a stronger tendency to support palladium. When prepared with low amounts of palladium (0.7 w/w, L-series), Pd@DHImel revealed indeed the presence of a significantly higher content of the metal, as inferred from the Pd/C atomic ratio equal to 0.08 ± 0.004 (Figure S5a), 1 order of magnitude higher than Pd@DHNmel (Pd/C = 0.008 ± 0.0004). On the other hand, no significant differences emerged from samples prepared with a higher quantity of Pd­(AcO)_2_ (7 w/w, H-series), with the Pd/C ratio varying from 0.33 ± 0.02 in the case of Pd@DHICAmel to 0.40 ± 0.02 in the case of Pd@DHNmel.

The inspection of the Pd 3d detailed XPS spectra revealed the presence of components accounting for different chemical states (Figure 6a–c). In each melanin hybrid sample, three components can be identified in the Pd 3d_5/2_ spectrum (Table S1), with the most intense between 335.7 and 336.3 eV ascribed to Pd(0).^34,35^ Two additional components at higher binding energy, identifiable as Pd­(II), are observed: the most intense, located at about 337.6 eV, can be due to Pd^2+^ species interacting with the melanin polymer at the level of the catechol functionalities^36^ and the additional Pd component at higher binding energy could be due to Pd­(II)-carboxylate species. From the comparison of Pd 3d spectra on the three hybrid samples, it clearly emerges that DHImel and DHICAmel are more efficient than DHNmel in promoting the reduction of Pd­(II) to Pd(0) for the presence of catechol functionalities that are more oxidizable than phenolic ones. This led to a low percentage of the Pd­(II) available in the Pd@DHImel and Pd@DHICAmel hybrids, with palladium mainly present and Pd(0) interacting with the carbon skeleton of the melanin polymer. Between DHICAmel and DHImel, the latter proved to be more efficient in promoting the reduction of Pd­(II) to Pd(0), exhibiting the highest component ascribed to the latter.

**Figure 6:**
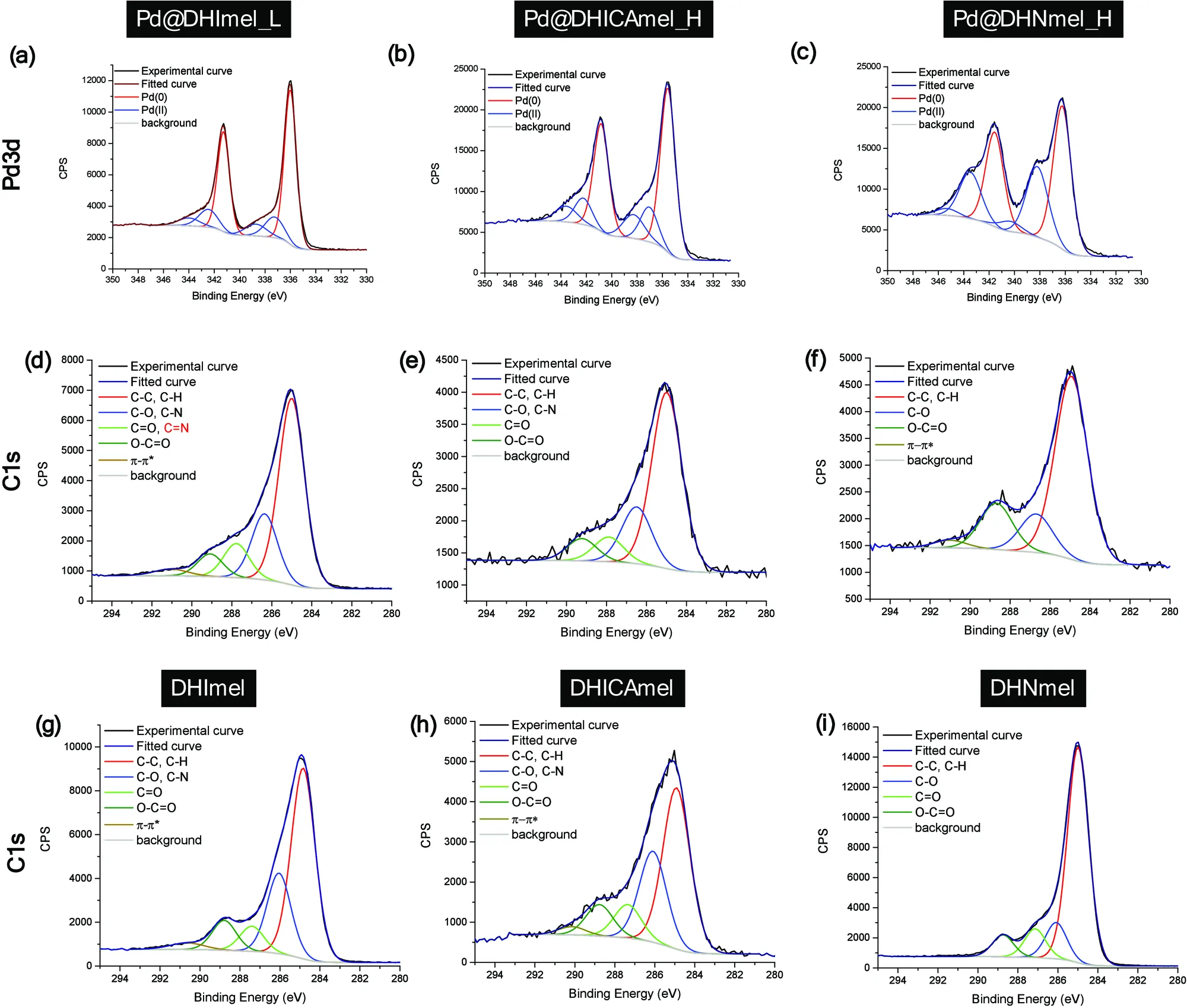
High-resolution X-ray photoelectron spectroscopy (XPS) signals: Pd 3d (a–c) and C 1s (d–f) of Pd@DHImel, Pd@DHICAmel, and Pd@DHNmel and C 1s (g–i) of DHImel, DHICAmel, and DHNmel.

The higher proneness of DHImel to undergo redox processes in comparison with DHICAmel and DHNmel is also observed from the analysis of C 1s XPS signals before (Figure 6g–i) and after (Figure 6d–f) the reaction with Pd. In the case of DHImel, upon Pd loading, a decrease of C–O and a simultaneous increase of the C=O component are evident (Table S1). Interestingly, this signal evolution is in line with the N 1s spectrum modification observed in DHImel after treatment with palladium (Figure S5b,c). Only in the case of Pd@DHImel, the appearance of a new component at about 399 eV, besides that at 400 eV due to pyrrolic −NH– bonds, is observed; this may be ascribed to the emergence of −C=N– bonds, likely due to the formation of quinoneimine units.

Overall, these data suggest that a redox exchange occurred preferentially between the metal and melanin polymers during the formation of the hybrid. A change in the redox state of the dihydroxyindole units is thus promoted by the interaction with palladium acetate, forcing the catechol moieties into *o*-quinone and quinoneimine assets (Figure 7A), accompanied by the reduction of Pd­(II) to Pd(0). As reported in the literature, this process is less favored in the case of DHICAmel and DHNmel due to steric hindrance and/or different redox properties, possibly explaining the peculiar DHImel behavior demonstrated by XPS analysis. Worthy of note is also the ratio between the areas of the =N– and −NH– components of the N 1s signal, equal to 0.21, which is very close to the ratio between the areas of the Pd^2+^ and Pd(0) components. This suggests that within the Pd@DHImel hybrid the residual Pd^2+^ may be almost chelated by indolic units in the quinonimine asset (Figure 7B).

**Figure 7:**
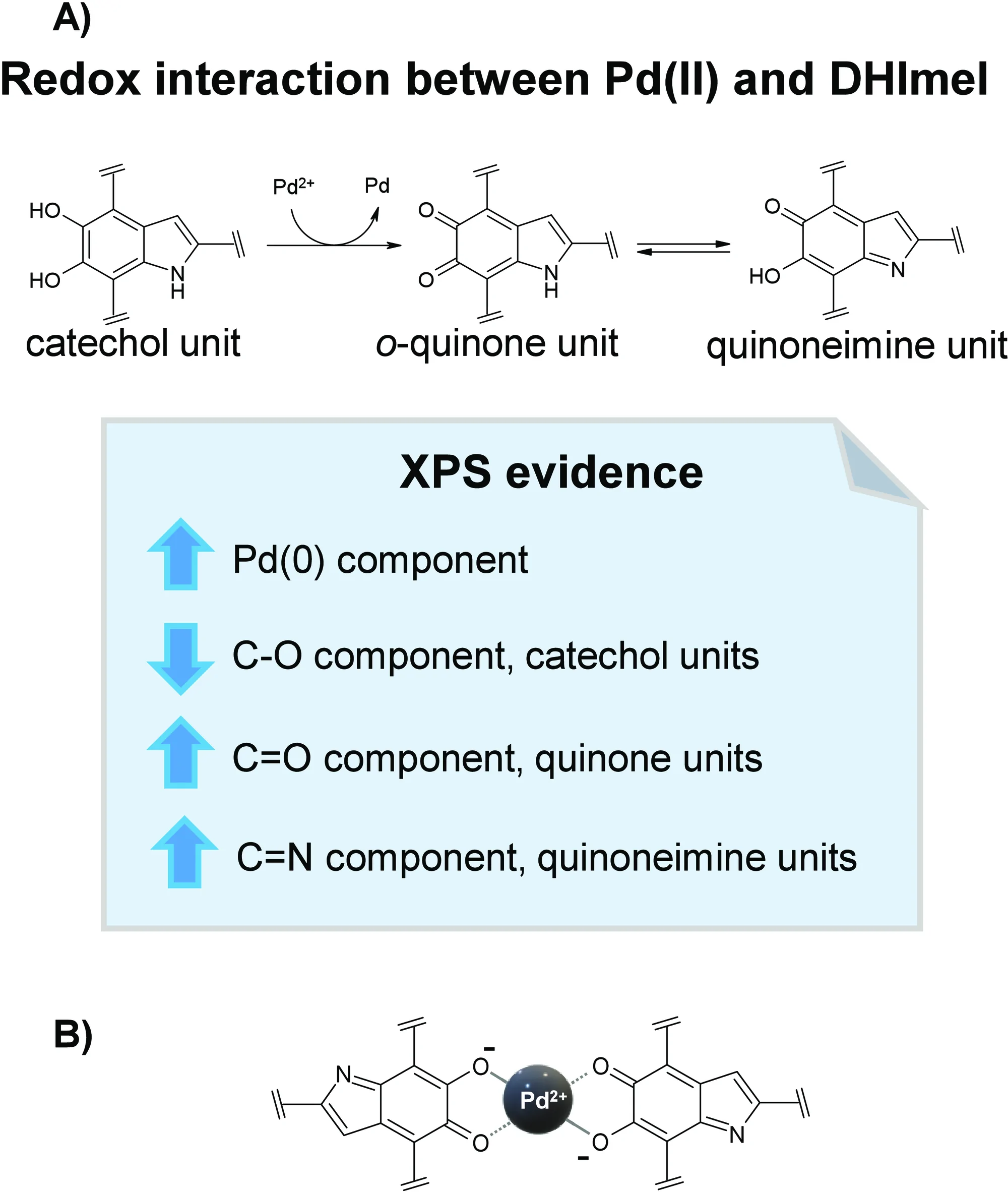
Interactions between Pd^2+^ and the dihydroxyindolic unit in DHImel via redox reaction (A) and chelation (B).

Evidence of a marked change of the redox state of the melanin polymer when forming the palladium hybrid also came from the ATR-FTIR spectra, revealing in the case of Pd@DHImel an appreciable change in the spectroscopic profiles with the appearance of C=O stretching bands at 1710 cm^–1^ (Figure S6). No significant changes were evident from Pd@DHICAmel and Pd@DHNmel, in good agreement with XPS data.

### 3.2 Catalytic Activity of Pd@Melanin Hybrids in the Sonogashira Cross-Coupling Reaction

The catalytic performance of the Pd@melanin hybrids was tested in the cross-coupling reaction of Sonogashira, which is one of the consolidated synthetic approaches to form new C–C bonds. Preliminary investigations were carried out on trimethylsilylacetylene (**1**) and *p*-iodoanisole (**2**), selected as model compounds, in the presence of triethylamine as the base and toluene as the solvent at 60 °C and under an argon atmosphere. Table 2 reports the data of the reactions carried out by using the different Pd@melanin hybrids previously obtained.

**Table 2:**
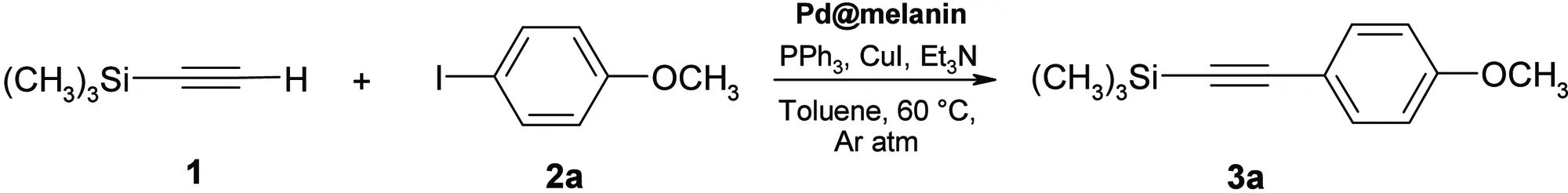
Pd@Melanin-Catalyzed Sonogashira Reaction of Trimethylsilylacetylene (**1**) with *p*-Iodoanisole (**2a**)­^a^

entry	Pd@melanin (mg)	palladium loading (mol %)	yields of **3a** (%)^b^
Pd@DHImel L-Series			
1	30	7.4	98
2	15	3.7	69
3	7.5	2.1	54
4	5	1.2	48
Pd@DHICAmel L-Series			
5	30	1.1	18
6	15	0.4	17
Pd@DHNmel L-Series			
7	30	0.5	25
8	15	0.3	18
Pd@DHImel H-Series			
9	30	24.2	100
10	15	12.1	99
11	5	4.2	99
Pd@DHICAmel H-Series			
12	30	25.8	99
13	15	13.2	93
14	5	4.2	91
Pd@DHNmel H-Series			
15	30	23.7	100
16	15	11.6	99
17	5	3.7	99

a Reaction conditions: *p*-iodoanisole (0.19 mmol), trimethylsilylacetylene (0.19 mmol), PPh_3_ (0.019 mmol), CuI (0.019 mmol), toluene/TEA 1:1 (v/v), 60 °C, 30 min, argon atm.

b Yields estimated by ^1^H NMR analysis.

All the hybrids exhibit a catalytic activity that may vary depending on the palladium loading and on the amount of catalyst used. In general, the catalysts prepared with a higher quantity of palladium (H-series) led to the formation of Sonogashira product **3a** with yields that are close to 100%, whereas the Pd@melanin hybrids of the L-series proved to be less effective, with only Pd@DHImel affording **3a** in high yields. No product formation was observed in the absence of the Pd@melanin catalyst or when using only the melanin polymer. The yields of formation of **3a** proved not to be affected by the type and amount of catalyst used in the case of the hybrids from the H-series, whereas a trend can be appreciated in the case of the hybrids from the L-series. In particular, Pd@DHImel and Pd@DHNmel proved to be more efficient than Pd@DHICAmel and higher yields of **3a** were obtained when using a higher quantity of the catalysts. The kinetic analysis revealed that the highest TOF values were obtained when using Pd@melanin catalysts of the L-series (Table S2).

Through a comparative analysis of the yields of **3a** as a function of the amount of catalyst/palladium loading, we extrapolated the best performing conditions for further analyses. In particular, in the case of DHImel, the palladium hybrid of the L-series was selected (amount of catalyst: 15 and 5 mg), whereas for both DHICAmel and DHNmel, the palladium hybrids of the H-series were selected (amount of catalyst: 15 mg).

To test the versatility of the Pd@melanin catalysts, we have carried out reactions by varying the nature of the alkynyl derivative, from an aliphatic alkyne (**1**) to an aromatic one (phenylacetylene, **4**), and of the aryl iodide; in particular, in this latter case, we have investigated the effect of the substituents on the selected aryl iodide.

The use of an aromatic alkyne resulted in a significant increase in the reaction yields of the cross-coupling product, as is evident from the data in Table 3. This may be ascribed to the well-known ability of melanin to establish efficient π-π stacking interactions with aromatic compounds such as drugs or pollutants.^37,38^ The interaction of melanin with the aryl iodide first and with the copper acetylide then may represent another peculiar advantage offered by the Pd@melanin hybrids in the mechanism of the Sonogashira reaction (Figure 8). By this way, phenylacetylene may have been bound to the melanin polymer more tightly than trimethylsilylacetylene (Figure 8, step 4), determining a faster cross-coupling.

**Table 3:**
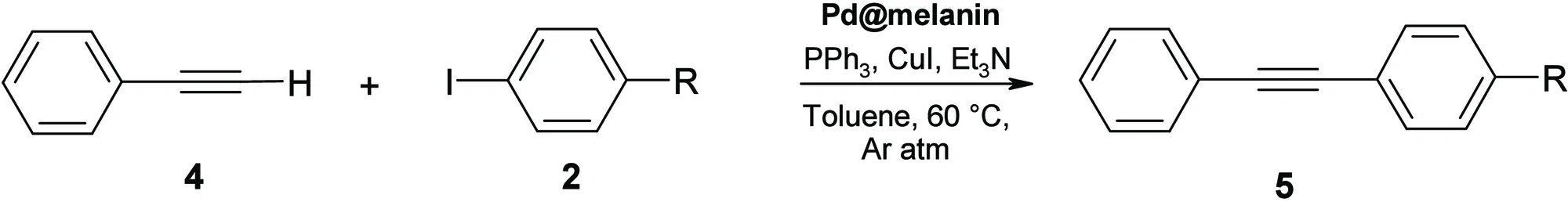
Pd@Melanin-Catalyzed Sonogashira Reaction of Phenylacetylene (**4**) with Aryl Iodide: Effect of the Substituent^a^

entry	Pd@melanin hybrid (H- or L-series)	amount of Pd@melanin (mg)	palladium loading (mol %)	yields (%)^b^	
**2a**, R = OCH_3_					
1	Pd@DHImel (L)	15	3.7	**5a**	100
2	Pd@DHImel (L)	5	1.2	**5a**	80
3	Pd@DHICAmel (H)	15	13.2	**5a**	89
4	Pd@DHNmel (H)	15	11.6	**5a**	100
**2b**, R = CH_3_					
5	Pd@DHImel (L)	15	3.7	**5b**	100
6	Pd@DHImel (L)	5	1.2	**5b**	85
7	Pd@DHICAmel (H)	15	13.2	**5b**	74
8	Pd@DHNmel (H)	15	11.6	**5b**	100
**2c**, R = NO_2_					
9	Pd@DHImel (L)	15	3.7	**5c**	100
10	Pd@DHImel (L)	5	1.2	**5c**	100
11	Pd@DHICAmel (H)	15	13.2	**5c**	45
12	Pd@DHNmel (H)	15	11.6	**5c**	100

a Reaction conditions: aryl iodide (0.19 mmol), phenylacetylene (0.19 mmol), PPh_3_ (0.019 mmol), CuI (0.019 mmol), toluene/TEA 1:1 (v/v), 60 °C, 30 min, argon atm.

b Yields estimated by ^1^H NMR analysis.

**Figure 8:**
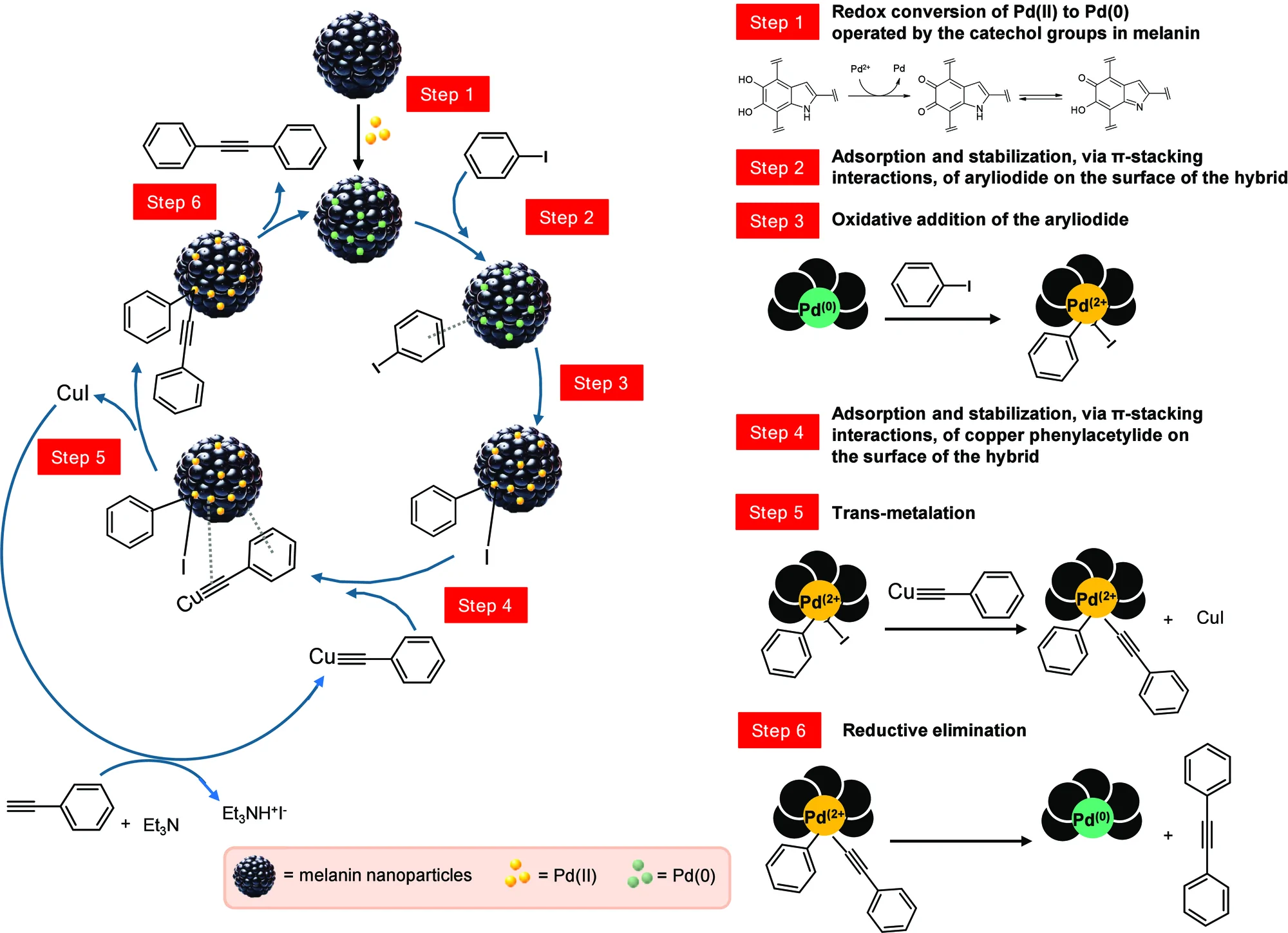
Catalytic cycle proposed for the Sonogashira cross-coupling reaction mediated by Pd@melanin hybrids.

In another set of experiments, we have also investigated the effect of the substituents on aryl iodide **2** and the role of the halide. In the first instance, we have selected *p*-iodoanisole (**2a**) and *p*-iodotoluene (**2b**), with electron releasing substituents, and *p*-nitroiodobenzene (**2c**) with an electron withdrawing substituent. The data reported in Tables 3 and 4 show a different effect, depending on the type of alkyne selected. In the case of phenylacetylene, the yields of formation of product **5** proved to be quite high regardless of the nature of the substituent on the aryl iodide (Table 3). However, when trimethylsilylacetylene was used as the starting reagent, the slow process allowed for a stricter effect of the substituents, with high yields recorded in the case of the activated **2a** and **2b**. Unexpectedly, the reaction yields obtained in the case of **2c** proved to be quite low (10–50%); this may depend on the interactions that the melanin polymers establish with the polar nitro-group in **2c**, partially suppressing its activating effect (Table 4).

**Table 4:**
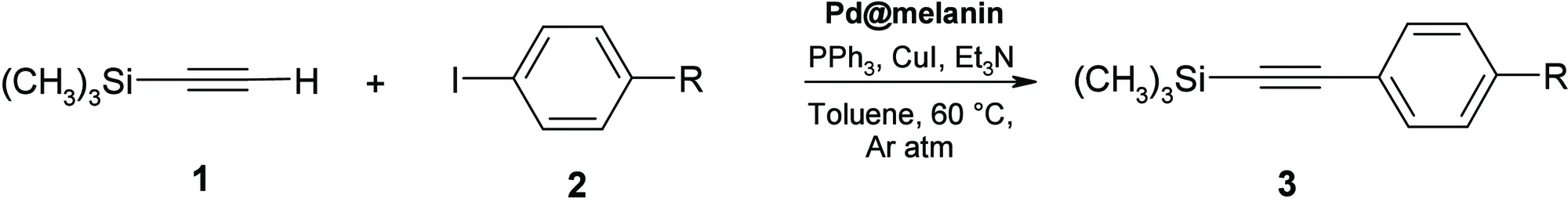
Pd@Melanin-Catalyzed Sonogashira Reaction of Trimethylsilylacetylene (**1**) with Aryl Iodide: Effect of the Substituent^a^

entry	Pd@melanin hybrid (H- or L-series)	amount of Pd@melanin (mg)	palladium loading (mol %)	yields (%)^b^	
**2a**, R = OCH_3_					
1	Pd@DHImel (L)	15	3.7	**3a**	69
2	Pd@DHImel (L)	5	1.2	**3a**	48
3	Pd@DHICAmel (H)	15	13.2	**3a**	93
4	Pd@DHNmel (H)	15	11.6	**3a**	99
**2b**, R = CH_3_					
5	Pd@DHImel (L)	15	3.7	**3b**	65
6	Pd@DHImel (L)	5	1.2	**3b**	42
7	Pd@DHICAmel (H)	15	13.2	**3b**	82
8	Pd@DHNmel (H)	15	11.6	**3b**	91
**2c**, R = NO_2_					
9	Pd@DHImel (L)	15	3.7	**3c**	53
10	Pd@DHImel (L)	5	1.2	**3c**	23
11	Pd@DHICAmel (H)	15	13.2	**3c**	11
12	Pd@DHNmel (H)	15	11.6	**3c**	37

a Reaction conditions: aryl iodide (0.19 mmol), trimethylsilylacetylene (0.19 mmol), PPh_3_ (0.019 mmol), CuI (0.019 mmol), toluene/TEA 1:1 (v/v), 60 °C, 30 min, argon atm.

b Yields estimated by ^1^H NMR analysis.

To test the substrate scope of the Pd@melanin catalyst, we checked the feasibility of the reaction conditions on other arylhalides (Table 5). As reported in Table 5, only the highly activated 2,4-dinitrochlorobenzene and, to a lesser extent, the 4-bromoacetophenone led to the formation of the desired product **5**, suggesting a good degree of selectivity of the catalyst. With poorly activated aryl chlorides the Sonogashira cross-coupling proved to be completely suppressed and only the product of the Glaser coupling was observed.

**Table 5:**
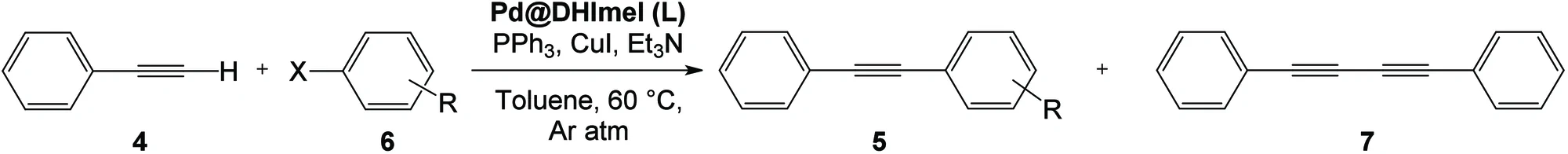
Pd@Melanin-Catalyzed Sonogashira Reaction of Phenylacetylene (**4**) with Other Aryl Halides^a^

entry	halide (X)	R	yields (%)^b^			
1	Cl	4-OMe (**6a**)	**5a**	n.d.	**7**	99
2	Cl	4-Me (**6b**)	**5b**	n.d.	**7**	98
3	Cl	2,4-dinitro (**6d**)	**5d**	41	**7**	n.d.
4	Br	4-C(O)Me (**6e**)	**5e**	8	**7**	n.d.

a Reaction conditions: Pd@DHImel (L-series, 15 mg, Pd loading 3.7 mol %), aryl halide (0.19 mmol), phenylacetylene (0.19 mmol), PPh_3_ (0.019 mmol), CuI (0.019 mmol), toluene/TEA 1:1 (v/v), 60 °C, 60 min, argon atm.

b Estimated by ^1^H NMR analysis, taking into account the final mass of the crude after work up.

In summary, among the melanin types tested in this study, DHImel proved to be the most effective, with its high affinity for palladium allowing for the preparation of hybrids with different loading, and with the most efficient catalytic activity leading to high yields of reaction with the lowest metal loading. These data proved to be quite interesting especially if considered into a more general panorama where the most common palladium catalysts typically used for promoting the Sonogashira cross-coupling reactions (i.e., Pd/C, Pd­(PPh_3_)_4_, Pd­(OAc)_2_, and PdCl_2_(PPh_3_)_2_) proceeded with lower yields, in the range 40–70% with respect to 80% obtained with Pd@DHImel (L) (Table 3, entry 2) with equal quantity of palladium used (Table S5).

The impact of the Pd@DHImel catalyst was also very striking if compared with the performances of other palladium catalysts supported on natural polymers (Table 6). With comparable reaction yields obtained, the Pd@DHImel catalyst emerged as a valuable choice because it does not require the chemical manipulation of the natural support and or harsh reaction conditions (i.e., temperature and reaction time) to operate the cross-coupling reaction.

**Table 6:** Sonogashira-Cross Coupling Reactions Catalyzed by Pd Ions Supported on Natural Polymers

entry	support	Pd loading (mol %)	reagents	reaction conditions	yields (%)	reference
1	ethylenediamine functionalized cellulose	0.4	*p*-iodoanisole phenylacetylene	H_2_O, CuI, K_2_CO_3_	95	^39^
				100 °C, 7 h		
2	cross-linked chitosan hybrid nanofibers	7	*p*-iodotoluene phenylacetylene	DMA/glycol, K_2_CO_3_	84	^40^
				110 °C, 5 h		
3	cellulose	1.9	*p*-iodoanisole phenylacetylene	DMF, TEA	98	^41^
				120 °C, 24 h		
4	phosphinite functionalized agarose	0.05	*p*-iodoanisole phenylacetylene	DMA, DABCO	75	^42^
				50 °C, 24 h		
5	chitosan-based montmorillonite	0.3	*p*-iodoanisole phenylacetylene	DMSO, KOAc	88	^43^
				110 °C, 5 h		
6	pectin	0.28	iodobenzene phenylacetylene	DMF, KOAc	100	^44^
				100 °C, 25 min		
7	silk fibroin	0.25	*p*-iodotoluene phenylacetylene	H_2_O/EtOH, TEA	61	^28^
				90 °C, 18 h		
8	DHImel	1.2	*p*-iodoanisole phenylacetylene	toluene, TEA	80	this work
				60 °C, 1 h		

To further check the sustainability of this well-performing new catalyst hybrid, finally we investigated the stability of the Pd@DHImel hybrid and the possibility of reusing it for multiple Sonogashira reactions. As reference, we have selected the Sonogashira reaction of phenylacetylene with *p*-iodonitrobenzene (Table 3, entry 9) that has been carried out repeatedly with the same Pd@DHImel catalyst for up to 10 cycles. After each cycle, the catalyst has been recovered, and after extensive washing with organic solvents, it has been reused for the next reaction cycle. ICP-MS analyses were carried out on each reaction mixture, confirming that no metal leaching occurred. ^1^H NMR analyses showed that the reaction yields were quite high (>85%) up to eight cycles (Figure 9), thus reinforcing the possibility to use Pd@DHImel hybrids as sustainable catalysts. Starting from the ninth cycle, a substantial reduction of the metal loading was observed (XPS and HR-TEM evidence, Figure S7), probably due to the mechanical detachment induced by the repeated washing needed to reuse the catalyst. However, the XPS data revealed no change in the distribution of the palladium components.

**Figure 9:**
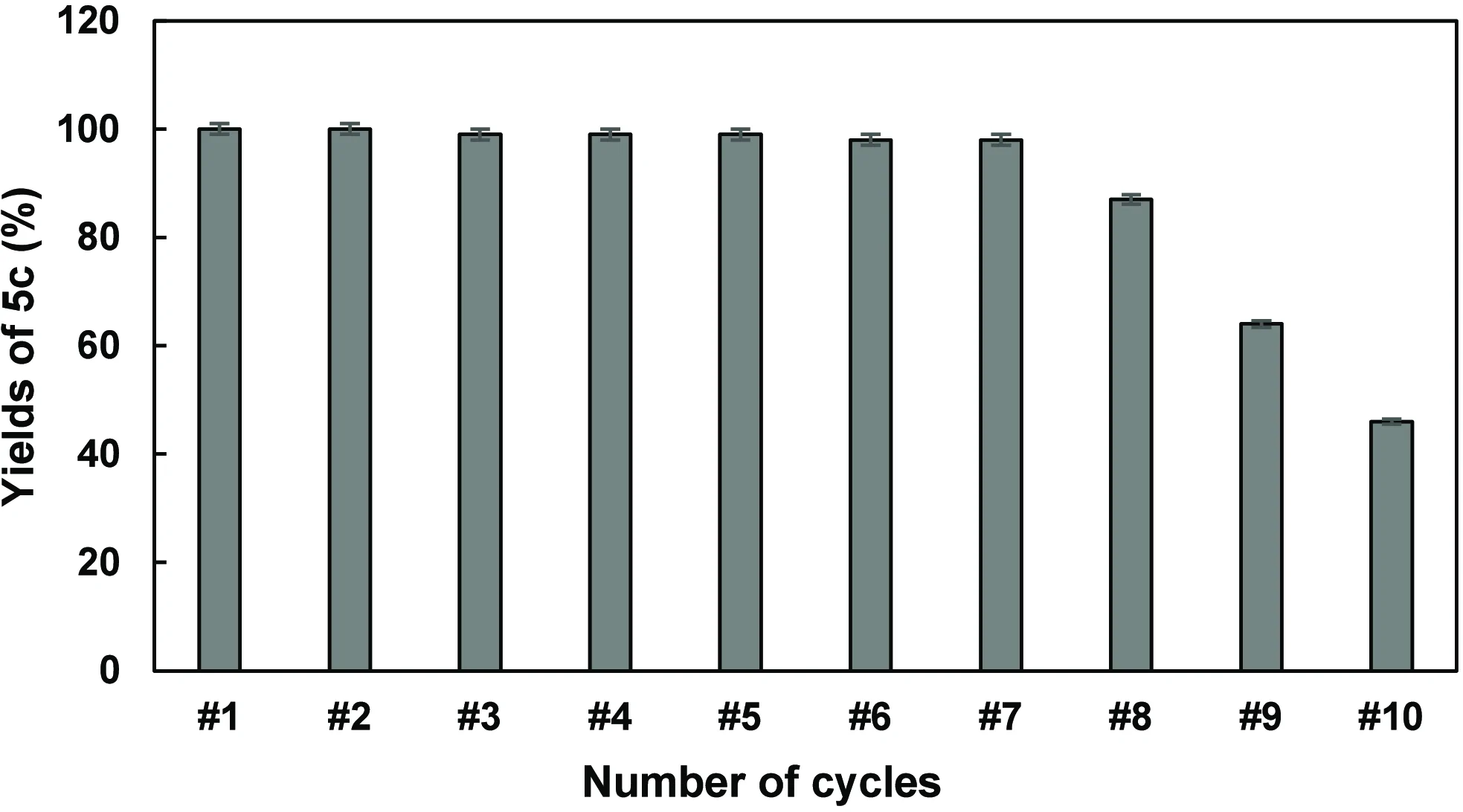
Recycling tests of Pd@DHImel promoted the Sonogashira cross-coupling reaction. Reaction conditions are reported in Table 3, entry 9. Data are reported as the mean of three separate experiments ± SD.

## 4 Conclusion

The pressing challenges of climate change and resource depletion demand a redefinition of chemical processes in terms of sustainability and environmental responsibility. Within this context, catalysis has undergone a profound transformation, driven by the integration of green principles and advanced materials design. Among emerging candidates, melanin represents a unique bioinspired platform that unites structural complexity, redox activity, and a strong metal-chelating ability. In this study, these intrinsic features were exploited to develop melanin-supported palladium catalysts that operate under mild, sustainable conditions, exemplifying how biologically derived materials can serve as efficient and robust systems for modern heterogeneous catalysis. Following a structure–property relationship approach, we have characterized the melanin hybrid materials and tested their catalytic performance in promoting the Sonogashira cross-coupling reaction.

What emerged is that melanin behaves as an active material that is able not only to bind Pd­(II) ions but also to promote the simultaneous reduction to Pd(0). This mechanism of activation of palladium proved to be more favored in the case of DHImelanin thanks to the high number of catechol moieties that can be oxidized, forming highly delocalized quinone species, and to the structural asset that favors the formation of highly conjugated planar extended quinone units. The Pd@DHImel hybrid material exhibited (1) high Pd loading values, achieved even when low amounts of Pd­(AcO)_2_ were used to prepare it; (2) a homogeneous distribution of the metal over the surface of the melanin polymer, forming aggregates with an average dimension of 8 nm; and (3) high stability and a reduced leaching effect, allowing for the possible reuse of the catalyst for up to eight cycles without affecting its performance.

Overall, these data delineate the enormous potential of melanin as an emergent class of sustainable heterogeneous catalysts, especially if we analyze their performances within the panorama of the recently developed Pd catalysts supported on natural polymers. In particular, not only are synthetic melanin pigments easily prepared under mild and sustainable conditions but, once obtained, they can be used to prepare the corresponding hybrids without any kind of functionalization process. Moreover, when such hybrids are used to promote the Sonogashira cross-coupling reaction, high yields of the desired product are obtained without using harsh conditions (i.e., high temperature and long reaction time). These premises set the stage for the development of a new generation of melanin catalysts, pointing out the relevant role played by DHImel and opening new applications of natural eumelanin pigments.

## Supplementary Material



## Notes

XRD, EDX, and ATR-FTIR spectra of melanin polymers and Pd@melanin hybrids; XPS spectra and data for melanin polymers and Pd@melanin hybrids; data for Pd@melanin catalyzed Sonogashira reactions (PDF)

## Abbreviations

ADF-STEM: annular dark field scanning transmission electron microscopy
ATR-FTIR: attenuated total reflectance Fourier transform infrared spectroscopy
DA: dopamine
DABCO: 1,4-diazabicyclo­[2.2.2]­octane
DHI: 5,6-dihydroxyindole
DHICA: 5,6-dihydroxyindole-2-carboxylic acid
DHN: 1,8-dihydroxynaphthalene
DMA: dimethylacetamide
DMF: dimethylformamide
DMSO: dimethyl sulfoxide
DOPA: 3,4-dihydroxyphenylalanine
EDX: energy dispersive X-ray spectroscopy
HRP: horseradish peroxidase
HR-TEM: high resolution transmission electron microscopy
ICP-MS: inductively coupled plasma mass spectrometry
NMR: nuclear magnetic resonance
SDD: silicon drift detector
TEA: triethylamine
TEM: transmission electron microscopy
TOF: turnover frequency
XPS: X-ray photoelectron spectroscopy
XRD: X-ray diffraction
XRPD: X-ray powder diffraction
