# Supplementary material for: A Straightforward Access to Sustainable and Reusable Melanin-Supported Palladium Catalysts: Characterization and Application in Sonogashira Cross-Coupling Reactions
Source: ACS Appl Mater Interfaces. 2026 Mar 31;18(14):20962–75. doi: 10.1021/acsami.5c23680 (PMC13730443; doi:10.1021/acsami.5c23680)
Supplement: Supplementary file 1 [file am5c23680_si_001.pdf]

Supporting Information

A Straightforward Access to Sustainable and Re-Usable Melanin Supported Palladium Catalysts: Characterization and Application in Sonogashira Cross-Coupling Reactions

Simone Potenti,[1,2] Pasquale Lioniello,[1] Elisabetta Mazzotta,[3] Nicoletta Ditaranto,[4] Evelina Liarou,[5] Noah Al-Shamery,[5,6] Pooi See Lee,[6] Teresa Sibillano,[7] Cinzia Giannini,[7] Cosimino Malitesta,[3] Paola Manini*[1,2]

[1]Department of Chemical Sciences, University of Naples Federico II, via Cintia 4, I-80126 Naples, Italy

[2]Bioelectronics Task Force, University of Naples Federico II, 80126 Naples, Italy

[3]Dipartimento di Scienze e Tecnologie Biologiche e Ambientali (Di.S.Te.B.A.), Università del Salento, via Monteroni, 73100 Lecce, Italy

[4]Dipartimento di Chimica and CSGI – Bari Unit, Università degli Studi di Bari Aldo Moro, Via Orabona 4, 70126 Bari, Italy

[5]Department of Chemistry, University of Warwick, Library Road, CV4 7AL Coventry, United Kingdom

[6]School of Materials Science and Engineering, Nanyang Technological University, Singapore, 50 Nanyang Ave, 639798 Singapore

[7]Institute of Crystallography, National Council of Research, CNR-IC, Via G. Amendola 122 - 70126, Bari, Italy

XRD spectra	S2-S3
EDX spectra	S4
XPS spectra and data	S5-S6
ATR-FTIR spectra	S7
Pd@melanin catalyzed Sonogashira reactions	S8-S10

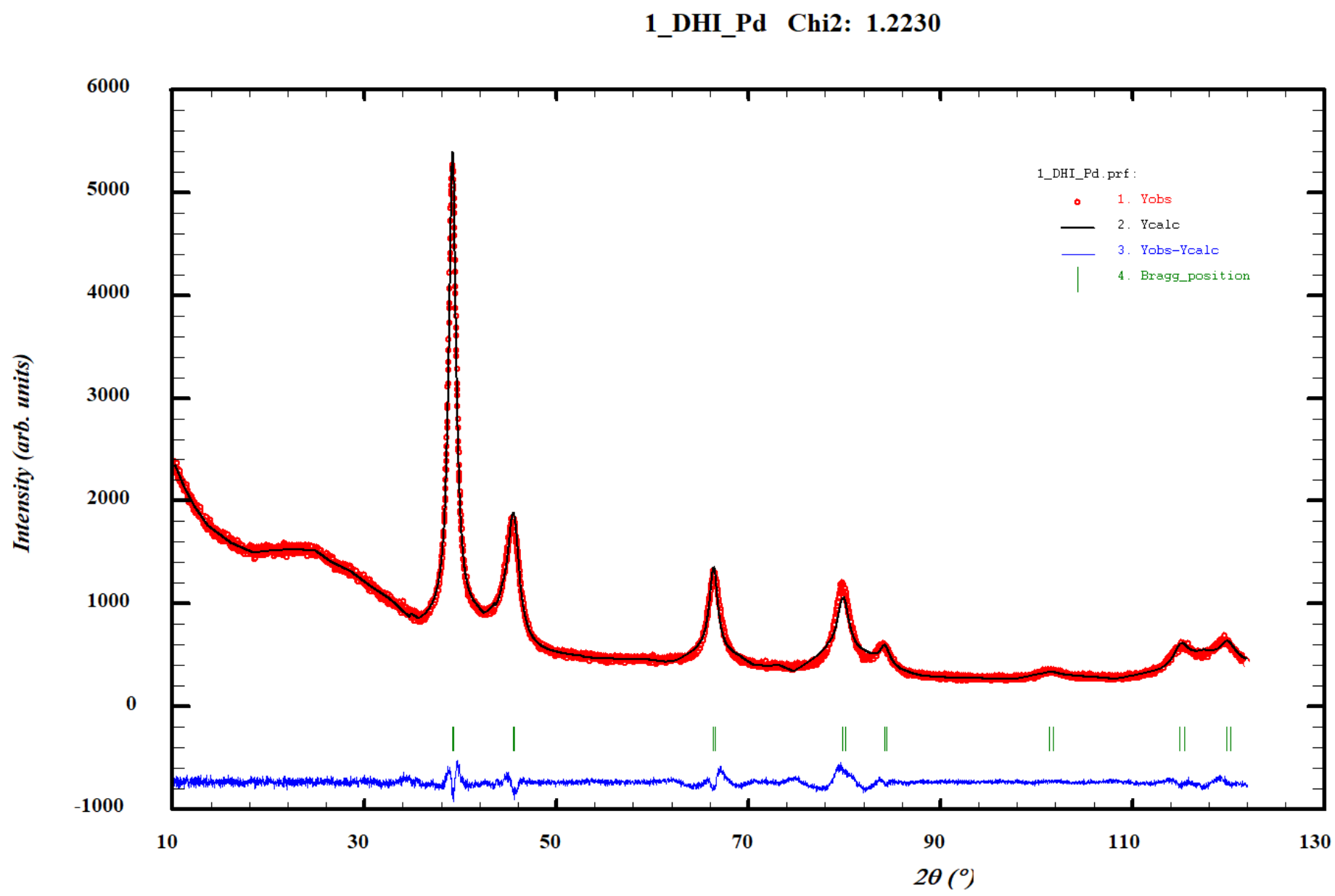

Figure S1. Rietveld refinement of the X-ray diffraction pattern for the Pd@DHImel sample. Experimental data (dots) are compared with the calculated profile (solid line), with the difference curve shown below. The refinement confirms the presence of metallic palladium in the face-centered cubic (f.c.c.) phase, yielding a lattice parameter of a = b = c = 3.98 Å and an apparent crystallite size of 64.7 ± 13.3 Å.

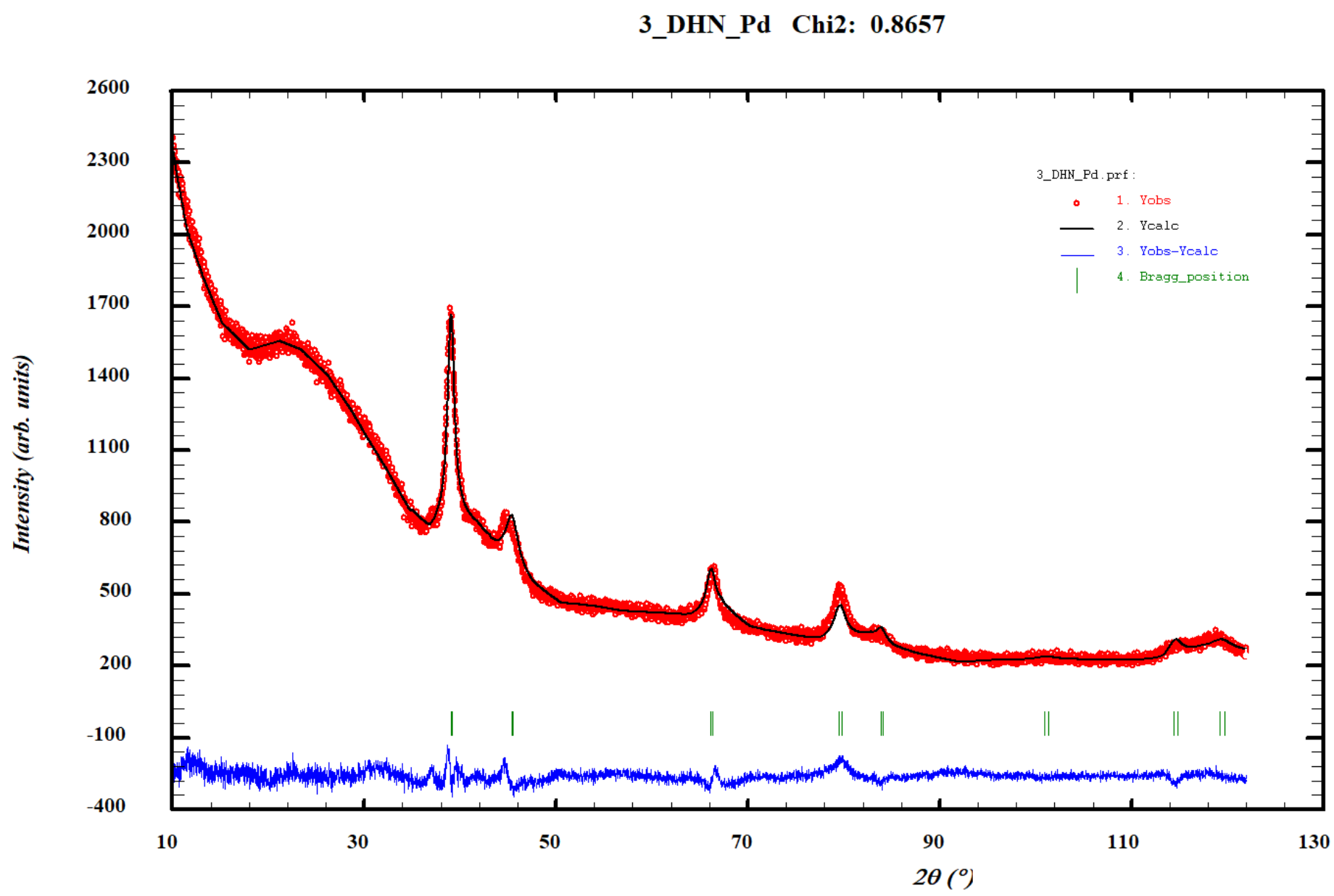

Figure S2. Rietveld refinement of the X-ray diffraction pattern for the Pd@DHNmel sample. Experimental data (dots) are compared with the calculated profile (solid line), with the difference curve shown below. The refinement confirms the presence of metallic palladium in the face-centered

cubic (f.c.c.) phase, yielding a lattice parameter of a = b = c = 3.99 Å and an apparent crystallite size of 59.9 ± 15.6 Å, consistent with nanocrystalline Pd.

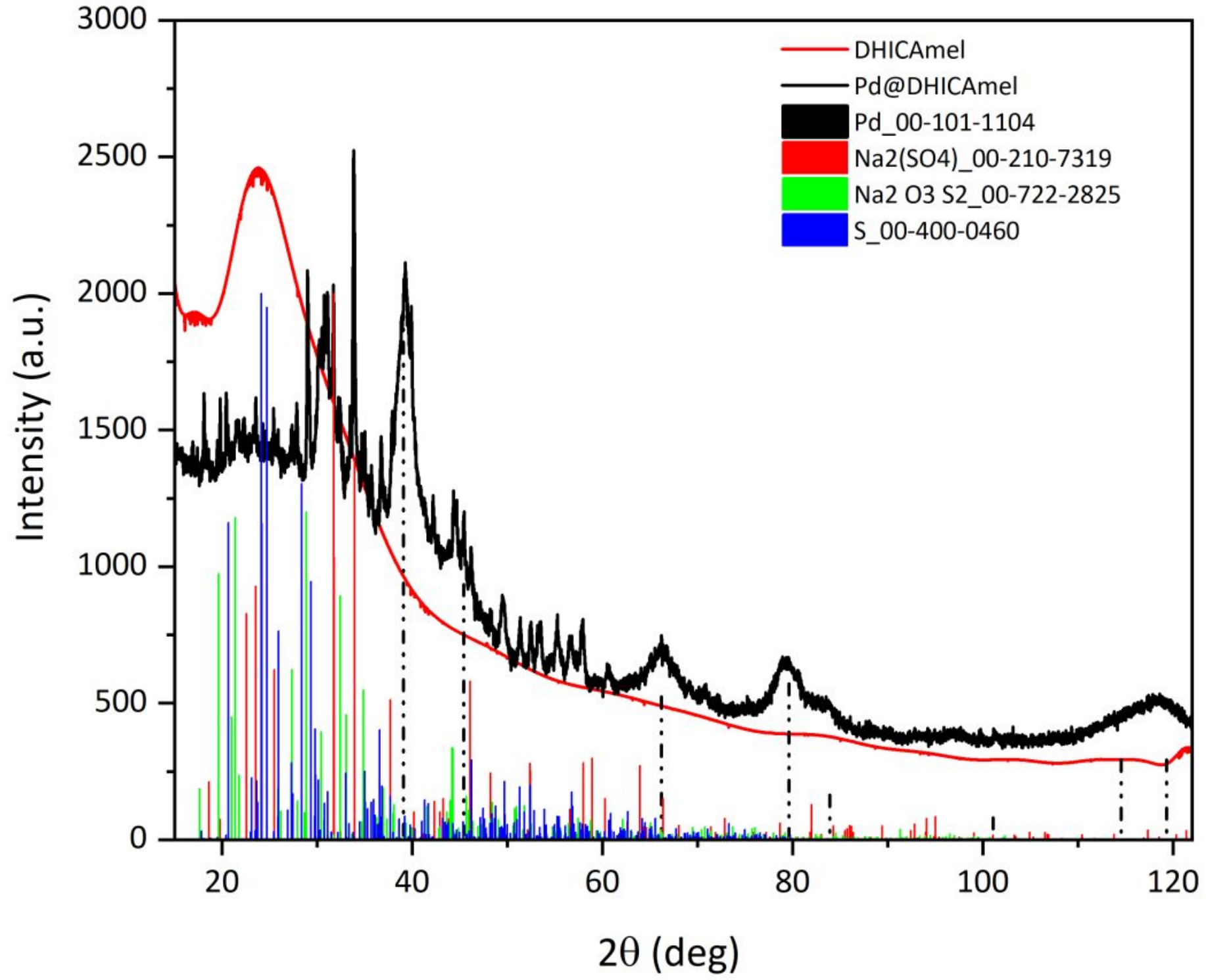

Figure S3. X-ray diffraction pattern of the Pd@DHICAmel sample, showing the qualitative phase analysis. Reflections corresponding to metallic palladium (f.c.c. phase, COD# 00-101-1104) are observed alongside additional peaks attributable to sodium sulfite (Na_2SO_3, COD# 00-210-7319), sodium thiosulfate ($Na_2S_2O_3$, COD# 00-722-2825), elemental sulfur (S, COD# 00-400-046060), and other possible minor crystalline phases.

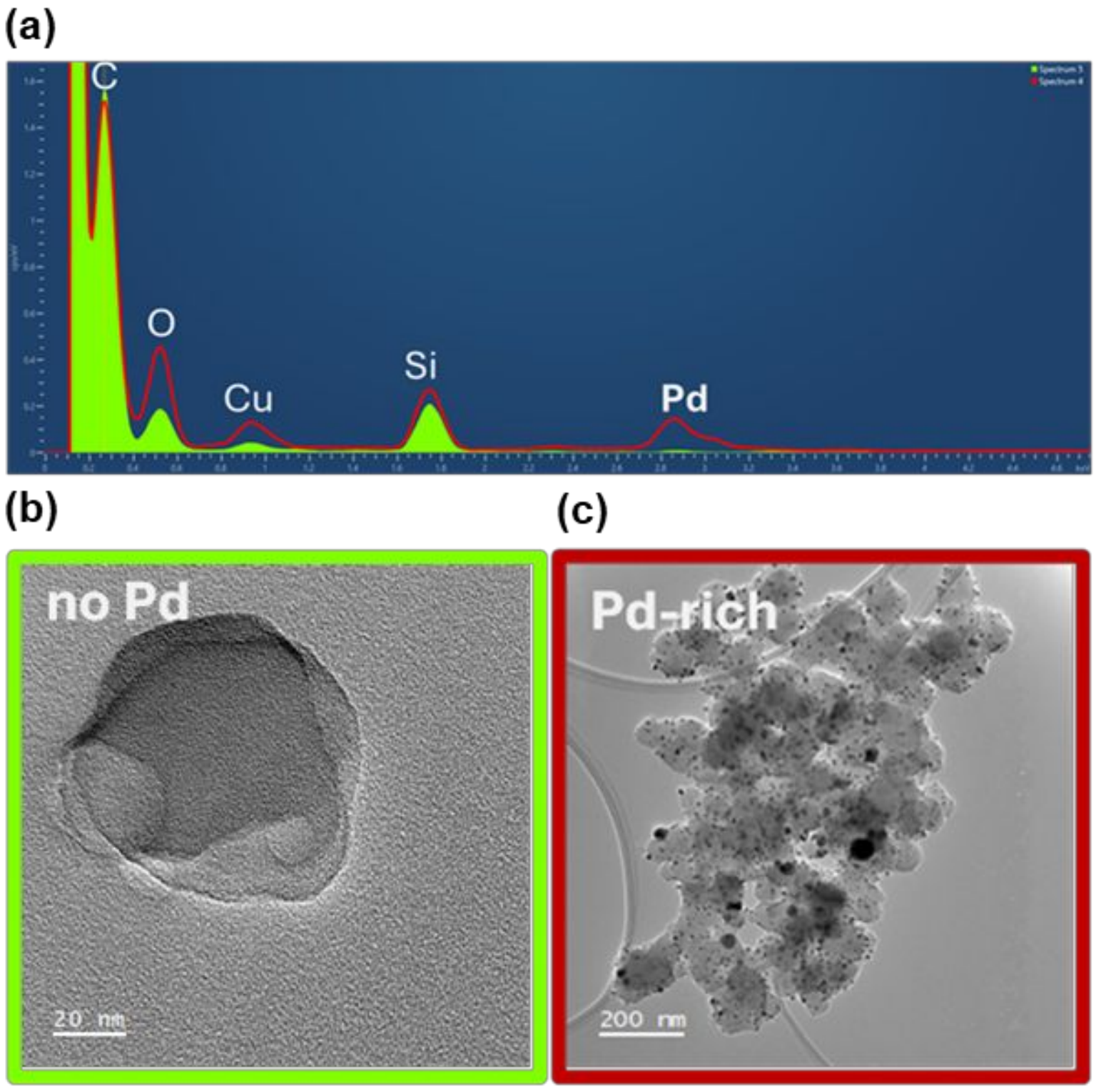

Figure S4. (a) comparison of the EDX spectra of the Pd@DHImel L-series taken from palladium-free (green trace and image b) and palladium-rich (red trace and image c) regions.

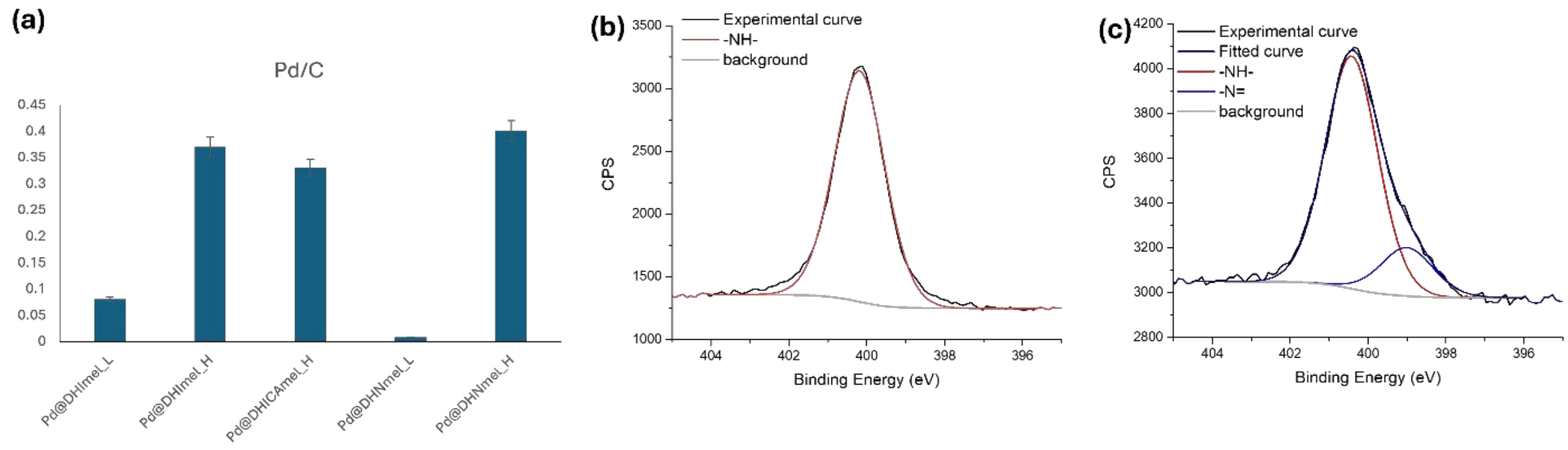

Figure S5. (a) Pd/C atomic ratio on different tested hybrid samples. Comparison of N *1s* signal of DHImel (b) and Pd@DHImel_L (c).

Table S1. C *1s*, N *1s* and Pd $3d_{5/2}$ binding energies (eV) and relative area percentages (%) of the bare melanin polymers (DHImel, DHICAmel and DHNmel) and their relative Pd hybrids (Pd@DHImel, Pd@DHICAmel and Pd@DHNmel).

Pd@melanin hybrids	C *1s*			
	C-O, C-N		C=O	
	BE (eV)	Area (%)	BE (eV)	Area (%)
DHImel	286.0±0.0	24.1±1.78	287.4±0.3	7.5±0.92
DHImel_red	286.2±0.0	25.2±1.12	287.5±0.1	8.0±0.88
Pd@DHImel_L	286.4±0.1	20.8±1.02	287.8±0.2	10.7±0.85
Pd@DHImel_Lred	286.3±0.1	19.9±1.39	287.6±0.4	11.0±0.97
Pd@DHImel_H	285.8±0.1	27.8±3.97	287.2±0.1	17.1±2.33
DHICAmel	286.2±0.1	26.2±2.70	287.4±0.2	10.3±0.75
Pd@DHICAmel_H	286.4±0.2	18.7±7.82	287.7±0.5	9.5±2.79
DHNmel	286.0±0.2	10.1±1.98	287.1±0.3	8.0±0.98
DHNmel_red	286.8±0.0	16.3±1.33	287.9±0.1	4.6±0.79
Pd@DHNmel_L	286.5±0.1	13.2±1.14	287.4±0.2	7.0±0.83
Pd@DHNmel_Lred	286.6±0.1	15.2±1.35	287.6±0.4	8.1±0.99
Pd@DHNmel_H	286.7±0.0	14.4±1.55	-	-

Pd@melanin hybrids	N *1s*			
	-NH-		-N=	
	BE (eV)	Area (%)	BE (eV)	Area (%)
DHImel	400.2±0.1	100	-	-
DHImel_red	400.4±0.0	100	-	-
Pd@DHImel_L	400.4±0.1	82.8±2.00	399.0±0.1	17.2±0.55
Pd@DHImel_Lred	400.4±0.0	86.7±2.10	398.8±0.1	13.2±0.65
Pd@DHImel_H	400.9±0.1	46.5±2.78	399.7±0.0	42.2±0.91

Pd@melanin hybrids	Pd *3d*			
	Pd(0)		Pd(II)	
	BE (eV)	Area (%)	BE (eV)	Area (%)
Pd@DHImel_L	336.0±0.1	43.9±2.0	337.2±0.1	9.4±0.9
Pd@DHImel_Lred	335.9±0.0	41.7±2.6	337.3±0.0	10.8±0.9
Pd@DHImel_H	336.2±0.2	41.7±0.5	337.6±0.1	5.4±1.3
Pd@DHICAmel_H	335.7±0.1	41.3±1.1	337.2±0.2	13.0±2.5
Pd@DHNmel_L	336.0±0.1	41.0±1.9	337.9±0.1	16.9±0.5
Pd@DHNmel_Lred	335.9±0.0	35.6±2.4	337.5±0.1	14.7±0.9
Pd@DHNmel_H	336.3±0.0	35.3±1.8	338.3±0.1	21.6±0.9

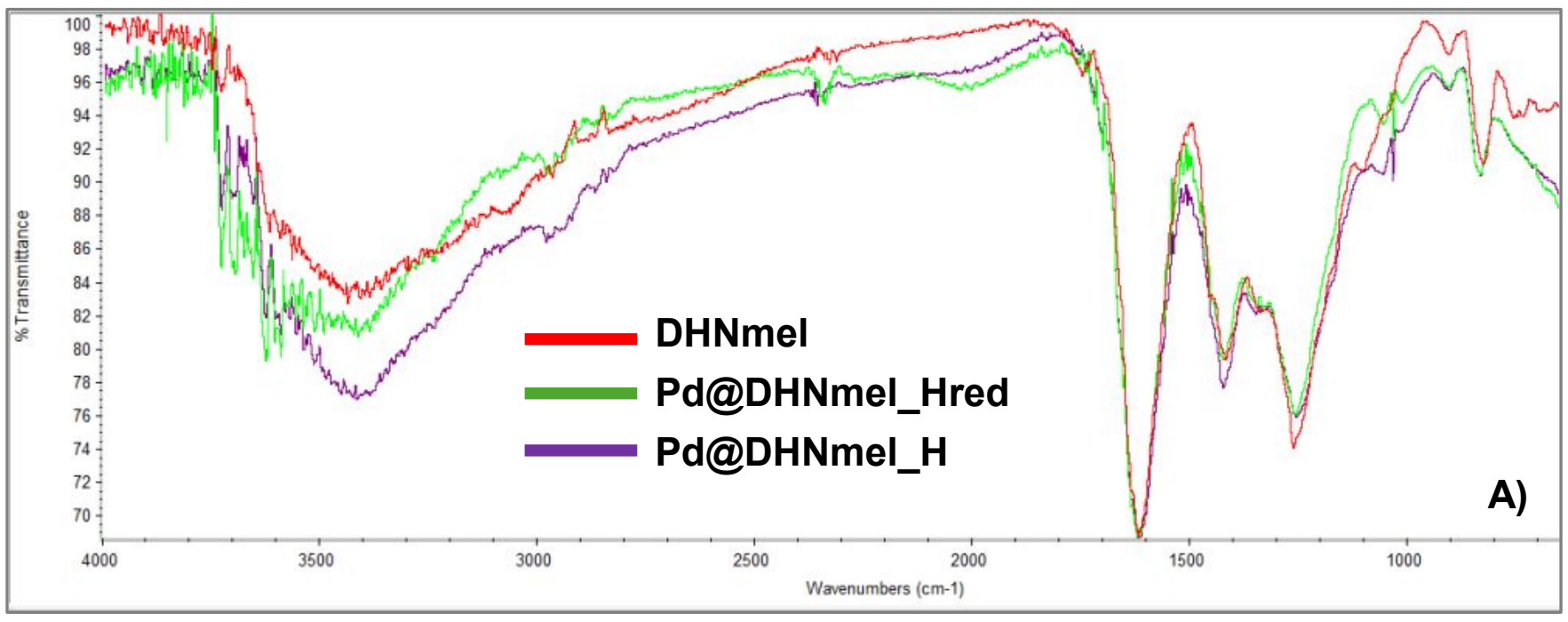

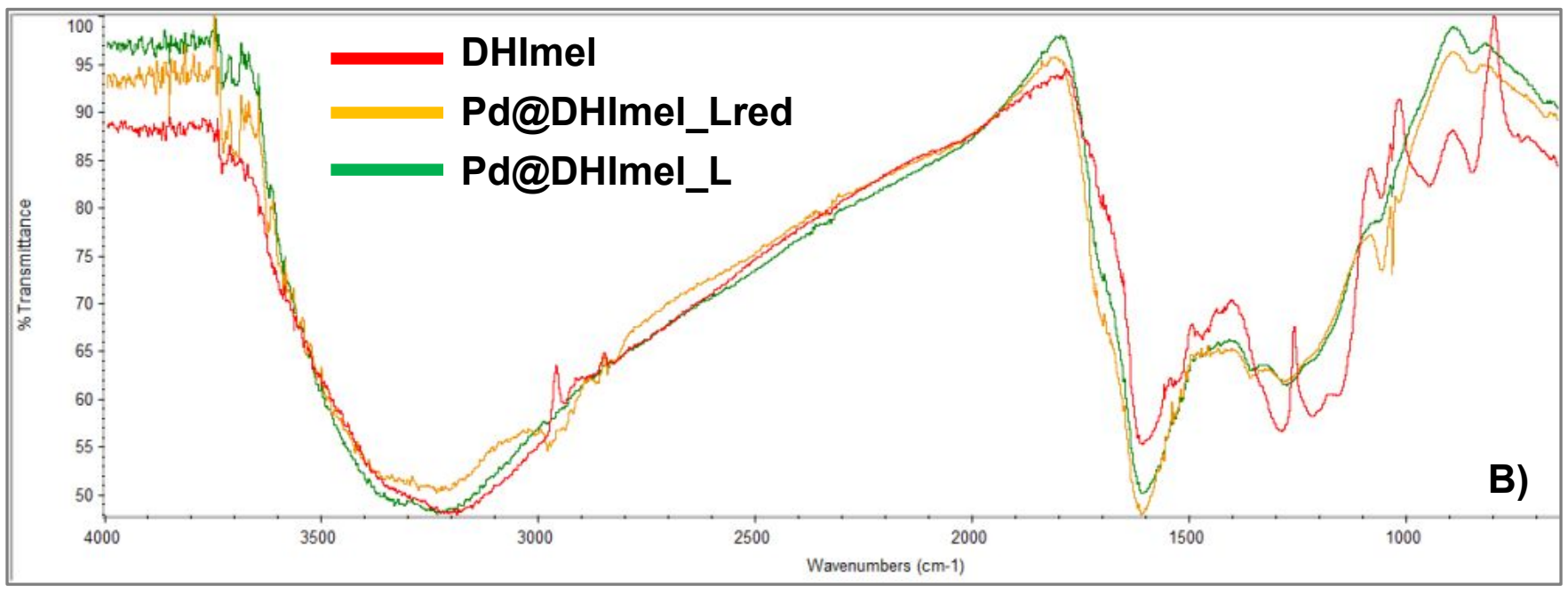

Figure S6. ATR-FTIR spectra. A) spectra from the Pd@DHImel_H and Pd@DHImel_Hred hybrids compared with the spectrum from the bare DHImel polymer; B) spectra from the Pd@DHNmel_L and Pd@DHNmel_Lred hybrids compared with the spectrum from the bare DHNmel polymer.

Table S2. Pd@melanin-catalyzed Sonogashira reaction of trimethylsilylacetylene (**1**) with *p*-iodoanisole (**2a**)

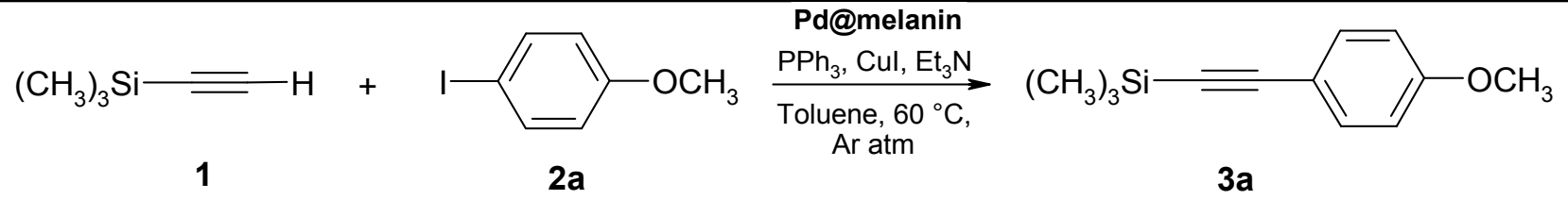

Entry	TOF (h^{-1})	Palladium loading (mol %)	Yields of 3a (%)
**Pd@DHImel L-series**			
1	26	7.4	98
2	37	3.7	69
3	51	2.1	54
4	80	1.2	48
**Pd@DHICAmel L-series**			
5	33	1.1	18
6	85	0.4	17
**Pd@DHNmel L-series**			
7	100	0.5	25
8	120	0.3	18
**Pd@DHImel H-series**			
9	8	24.2	100
10	16	12.1	99
11	47	4.2	99
**Pd@DHICAmel H-series**			
12	8	25.8	99
13	14	13.2	93
14	44	4.2	91
**Pd@DHNmel H-series**			
15	8	23.7	100
16	16	11.6	99
17	54	3.7	99

Table S3. Pd@melanin-catalyzed Sonogashira reaction of phenylacetylene (**4**) with aryl iodide: effect of the substituent.

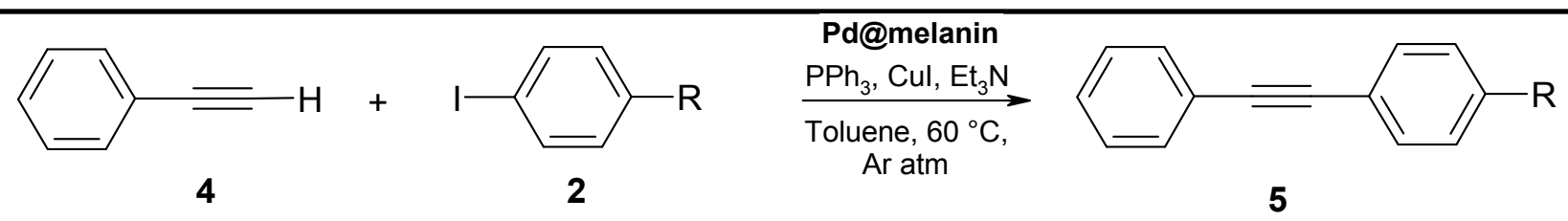

Entry	Pd@melanin hybrid (H- or L-series)	TOF (h^{-1})	Palladium loading (mol %)	Yields (%)	
**2a, R = OCH_3**					
1	**Pd@DHImel (L)**	54	3.7	**5a**	100
2	**Pd@DHImel (L)**	134	1.2	**5a**	80
3	**Pd@DHICAmel (H)**	14	13.2	**5a**	89
4	**Pd@DHNmel (H)**	16	11.6	**5a**	100
**2b, R = CH_3**					
5	**Pd@DHImel (L)**	54	3.7	**5b**	100
6	**Pd@DHImel (L)**	142	1.2	**5b**	85
7	**Pd@DHICAmel (H)**	12	13.2	**5b**	74
8	**Pd@DHNmel (H)**	16	11.6	**5b**	100
**2c, R = NO_2**					
9	**Pd@DHImel (L)**	54	3.7	**5c**	100
10	**Pd@DHImel (L)**	166	1.2	**5c**	100
11	**Pd@DHICAmel (H)**	8	13.2	**5c**	45
12	**Pd@DHNmel (H)**	16	11.6	**5c**	100

Table S4. Pd@melanin-catalyzed Sonogashira reaction of trimethylsilylacetylene (**1**) with aryl iodide: effect of the substituent.

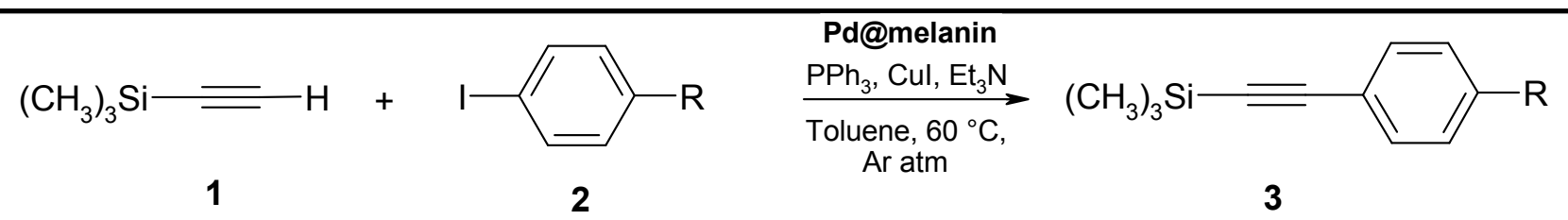

Entry	Pd@melanin hybrid (H- or L-series)	TOF (h^{-1})	Palladium loading (mol %)	Yields (%)	
**2a, R = OCH_3**					
1	**Pd@DHImel (L)**	39	3.7	**3a**	69
2	**Pd@DHImel (L)**	80	1.2	**3a**	48
3	**Pd@DHICAmel (H)**	14	13.2	**3a**	93
4	**Pd@DHNmel (H)**	16	11.6	**3a**	99
**2b, R = CH_3**					
5	**Pd@DHImel (L)**	36	3.7	**3b**	65
6	**Pd@DHImel (L)**	70	1.2	**3b**	42
7	**Pd@DHICAmel (H)**	12	13.2	**3b**	82
8	**Pd@DHNmel (H)**	16	11.6	**3b**	91
**2c, R = NO_2**					
9	**Pd@DHImel (L)**	28	3.7	**3c**	53
10	**Pd@DHImel (L)**	38	1.2	**3c**	23
11	**Pd@DHICAmel (H)**	2	13.2	**3c**	11
12	**Pd@DHNmel (H)**	6	11.6	**3c**	37

Table S5. Pd-catalyzed Sonogashira reaction of phenylacetylene (**4**) with *p*-iodoanisole (**2a**)

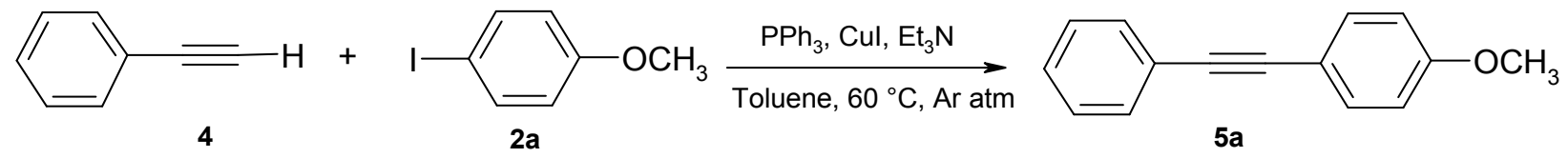

Entry	Pd catalyst	Palladium amount (mol %)	Yields of 5a (%)
1	Pd/C	1.2	60
2	$Pd(PPh_3)_4$	1.2	70
3	$Pd(OAc)_2$	1.2	40
4	$PdCl_2(PPh_3)_2$	1.2	63
5	-	-	-
6[a)]	-	-	-

a) DHImelanin was added (15 mg).

Table S6. Metal levels determined in Pd@DHImel_L, Pd@DHICAmel_L and Pd@DHNmel_L by ICP-MS analyses.

	**Palladium content (μg/mg of hybrid)**
Pd@DHImel_L	25
Pd@DHICAmel_L	5
Pd@DHNmel_L	2

A)

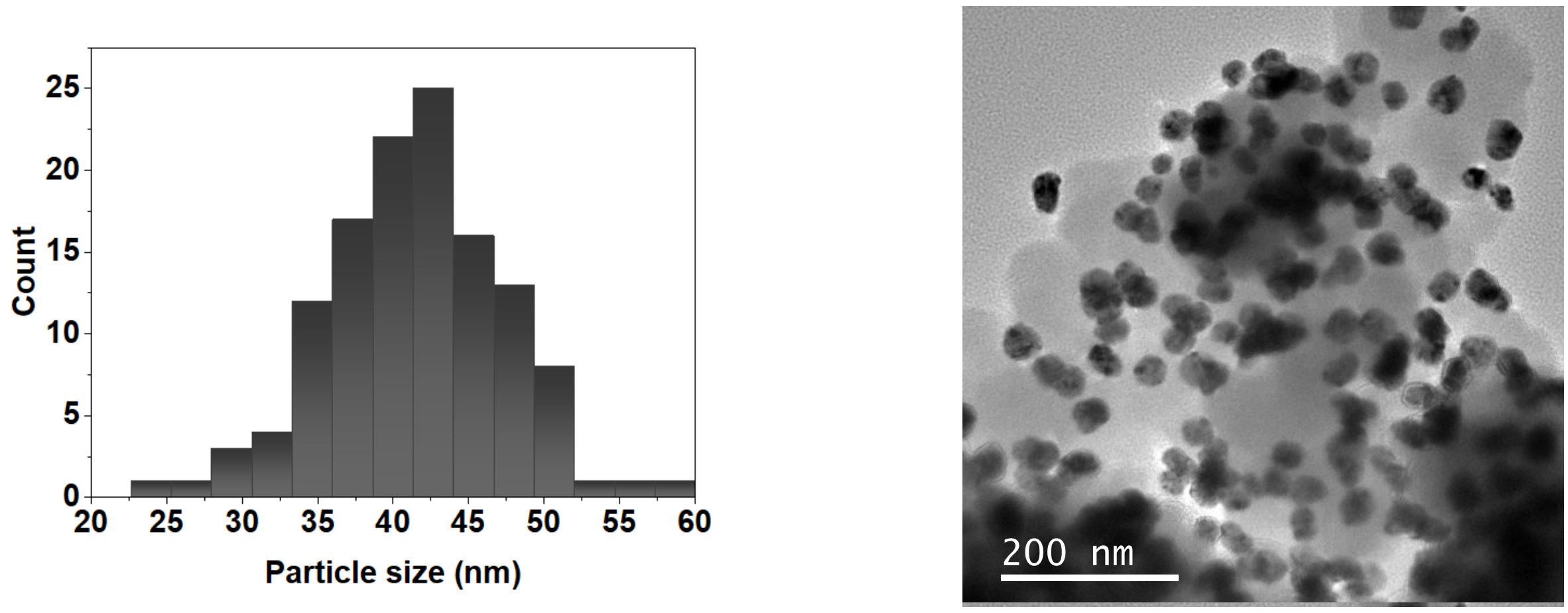

B)

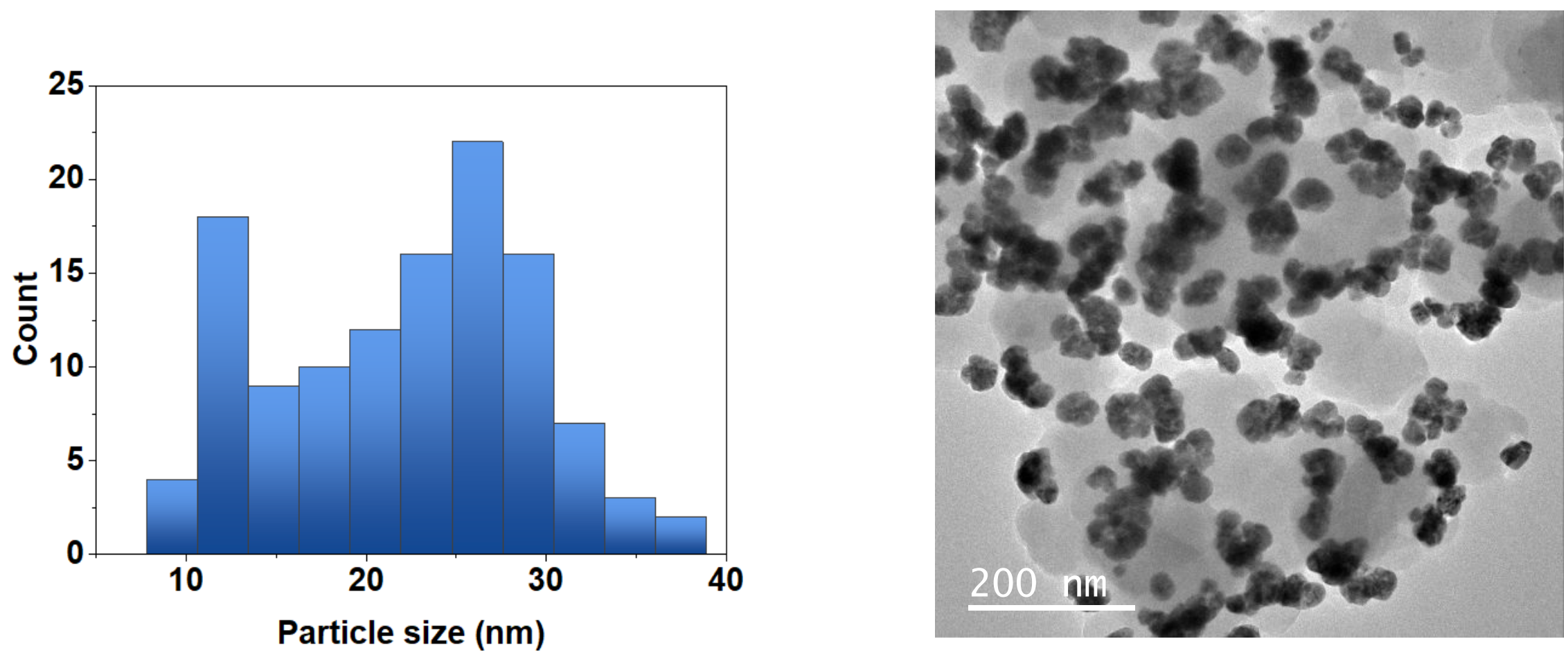

Figure S7. Pd@DHImel L-series: palladium average particle size of the catalyst (left) and HR-TEM images (right) before the use (A) and after eight catalytic cycles.
